# Integrated sample-handling and mounting system for fixed-target serial synchrotron crystallography

**DOI:** 10.1107/S2059798321001868

**Published:** 2021-04-19

**Authors:** Gabrielle Illava, Richard Jayne, Aaron D. Finke, David Closs, Wenjie Zeng, Shawn K. Milano, Qingqiu Huang, Irina Kriksunov, Pavel Sidorenko, Frank W. Wise, Warren R. Zipfel, Benjamin A. Apker, Robert E. Thorne

**Affiliations:** a Department of Chemistry and Chemical Biology, Cornell University, Ithaca, NY 14853, USA; b MiTeGen LLC, PO Box 3867, Ithaca, NY 14850, USA; c MacCHESS, Cornell University, Ithaca, NY 14853, USA; d Department of Molecular Biology and Genetics, Weill Institute for Cell and Molecular Biology, Cornell University, Ithaca, NY 14853, USA; e School of Applied and Engineering Physics, Cornell University, Ithaca, NY 14853, USA; f Meinig School of Biomedical Engineering, Cornell University, Ithaca, NY 14853, USA

**Keywords:** protein crystallography, serial crystallography, sample-delivery system, high throughput, sample support, serial synchrotron crystallography

## Abstract

Serial synchrotron crystallography (SSX) is enabling the efficient use of small crystals for structure–function studies of biomolecules and for drug discovery. An integrated SSX system has been developed comprising ultralow background-scatter sample holders suitable for room and cryogenic temperature crystallographic data collection, a sample-loading station and a humid ‘gloveless’ glovebox. The sample holders incorporate thin-film supports with a variety of designs optimized for different crystal-loading challenges. These holders facilitate the dispersion of crystals and the removal of excess liquid, can be cooled at extremely high rates, generate little background scatter, allow data collection over >90° of oscillation without obstruction or the risk of generating saturating Bragg peaks, are compatible with existing infrastructure for high-throughput cryocrystallography and are reusable. The sample-loading station allows sample preparation and loading onto the support film, the application of time-varying suction for optimal removal of excess liquid, crystal repositioning and cryoprotection, and the application of sealing films for room-temperature data collection, all in a controlled-humidity environment. The humid glovebox allows microscope observation of the sample-loading station and crystallization trays while maintaining near-saturating humidities that further minimize the risks of sample dehydration and damage, and maximize working times. This integrated system addresses common problems in obtaining properly dispersed, properly hydrated and isomorphous microcrystals for fixed-orientation and oscillation data collection. Its ease of use, flexibility and optimized performance make it attractive not just for SSX but also for single-crystal and few-crystal data collection. Fundamental concepts that are important in achieving desired crystal distributions on a sample holder via time-varying suction-induced liquid flows are also discussed.

## Abstract 2

A highly flexible, integrated system for preparing samples for serial synchrotron crystallography at room and cryogenic temperatures is described and evaluated. The system is compatible with the existing infrastructure for high-throughput crystallography at synchrotrons and addresses major issues in maximizing data quantity and quality from large numbers of small crystals.

## 1. Introduction   

As X-ray crystallography has passed its centenary (Bragg, 1912 ▸) and >133 000 macromolecular crystallographic structures have been deposited in the Protein Data Bank (https://www.rcsb.org/), the field of macromolecular crystallography (Rupp, 2009 ▸) continues to evolve. Protein crystallization remains a primary bottleneck (Gavira, 2016 ▸; McPherson & Gavira, 2014 ▸). Complementary techniques such as small-angle X-ray scattering (Tuukkanen *et al.*, 2017 ▸; Skou *et al.*, 2014 ▸; Hura *et al.*, 2009 ▸), NMR and cryo-EM (Cheng *et al.*, 2015 ▸; Lyumkis, 2019 ▸; Herzik *et al.*, 2019 ▸) avoid this bottleneck, but do so at the price of lower structural resolution, analyte size restrictions and temperature restrictions, respectively. One solution is serial crystallography (SX), the development of which began in the 1970s with the structural study of viral particles and has dramatically expanded in recent years as high-brilliance sources such as X-ray free-electron lasers (XFELs; Chapman *et al.*, 2011 ▸; Hunter *et al.*, 2014 ▸; Schlichting, 2015 ▸) and fourth-generation storage-ring sources (Eriksson *et al.*, 2014 ▸) have become available.

X-ray radiation damage limits the amount of useful diffraction data collected per unit crystal volume (Holton, 2009 ▸). Cooling crystals to ∼100 K reduces the damage rate by one to three orders of magnitude (Teng & Moffat, 2000 ▸; Warkentin *et al.*, 2014 ▸, 2017 ▸; Atakisi *et al.*, 2019 ▸), allowing a corresponding increase in the data collected per crystal. However, typical cooling rates may not capture the biologically relevant conformation of the protein (Fraser *et al.*, 2011 ▸; Keedy *et al.*, 2015 ▸), and the cryoprotectants added to prevent internal and external solvent from crystallizing can interfere with active sites and be mistaken for ligands in enzymology and drug discovery (Rupp, 2009 ▸; Deller & Rupp, 2015 ▸; Wlodawer *et al.*, 2018 ▸).

Serial crystallography addresses the radiation-damage issue by assembling a complete data set from partial data sets acquired from many crystals, where (using non-XFEL sources) the dose per crystal is strictly limited. A multiple-crystal approach was necessary prior to the widespread adoption of cryocrystallographic methods (Diederichs & Wang, 2017 ▸), especially in virus crystallography, and is essential in microcrystallography even at 100 K (Holton & Frankel, 2010 ▸). A key aspect of contemporary serial crystallography is maximizing the throughput and minimizing the cost per structure. Its potential impact extends well beyond microcrystallography to include pharmaceutical/biotechnological applications where crystal size is not an issue.

The unique characteristics of XFEL sources have driven innovation in SX sample-delivery systems and data-analysis methods. Many serial femtosecond crystallography (SFX) innovations have been adapted for serial synchrotron crystallography (SSX) at storage-ring (SR) light sources (Diederichs & Wang, 2017 ▸). ‘Diffraction before destruction’ at XFELs limits the useful exposure of each crystal to a single ∼10–100 fs X-ray pulse. This single pulse delivers a larger dose than is feasible (given radiation-damage limits) with SR sources, while producing nearly damage-free diffraction (Nass, 2019 ▸). An XFEL pulse containing 10^12^ photons with a full-width at half-maximum (FWHM) of 0.7 µm (a typical focused limit) or 5 µm (a more practical value) delivers a dose of ∼10^9^ Gy or ∼2 × 10^7^ Gy, respectively, compared with maximum tolerable doses at SR sources of 2 × 10^7^ Gy for cryocooled crystals and ∼0.5–5 × 10^5^ Gy for room-temperature (RT) crystals at 1.5–2 Å resolution (Teng & Moffat, 2000 ▸; Leal *et al.*, 2013 ▸; Warkentin *et al.*, 2014 ▸, 2017 ▸; Atakisi *et al.*, 2019 ▸). However, XFEL pulses do far more damage to crystal regions, and other crystals, outside the beam (Doak *et al.*, 2018 ▸; Wiedorn *et al.*, 2018 ▸). Their advantage in useful diffraction per unit crystal volume diminishes as the lateral crystal size grows beyond the beam size, and also because crystals within a radius of ∼25 µm or more of an exposed crystal may become unusable (Doak *et al.*, 2018 ▸). The orientation of the crystal during the femto­second exposure is fixed (and usually unknown), so most reflections are only partially recorded (Uervirojnangkoorn *et al.*, 2015 ▸). Indexing and structure-factor determination can require thousands of microcrystals.

Given the limited access to XFELs, most serial crystallo­graphy data is likely to be collected at SR sources (Diederichs & Wang, 2017 ▸). In SSX, crystals can be rotated during each (millisecond to second) exposure, reducing the reflection partiality and the number of diffraction frames and crystals required for structure determination (Wierman *et al.*, 2019 ▸). In one example, the numbers of crystals required for structure determination using no oscillation and using 1° and 3° oscillations per crystal were >3000, <500 and <300, respectively (Wierman *et al.*, 2019 ▸).

Sample-delivery systems for serial crystallography include both ‘moving-target’ and ‘fixed-target’ designs (Cheng, 2020 ▸; Martiel *et al.*, 2019 ▸; Zhao *et al.*, 2019 ▸). Moving-target designs flow or project crystals through the X-ray beam. Examples include LCP injectors (Weierstall *et al.*, 2014 ▸), gas dynamic virtual nozzles to generate microdrop or continuous jet streams (DePonte *et al.*, 2008 ▸; Nelson *et al.*, 2016 ▸; Wiedorn *et al.*, 2018 ▸), high-viscosity microstreams (Botha *et al.*, 2015 ▸) and electro-spinning injectors (Sierra *et al.*, 2012 ▸). In a hybrid design, a microfluidic mixer combines microcrystals with reaction buffer and deposits them onto moving Kapton tape for presentation to the beam, allowing time-resolved studies of reactions with timescales of seconds (Beyerlein *et al.*, 2017 ▸).

Fixed-target designs place crystals on supports that are rastered or stepped through the X-ray beam, either with fixed orientation (Hunter *et al.*, 2014 ▸), with oscillation (Zander *et al.*, 2015 ▸; Wierman *et al.*, 2019 ▸) or with helical scanning (Hasegawa *et al.*, 2017 ▸). Key aspects of current fixed-target serial microcrystallography, including the use of ultrathin substrates with arrays of holes and liquid removal by back-side blotting to minimize background scatter, were demonstrated 14 years ago using MiTeGen MicroMeshes, which were initially developed to enable data collection from hundreds of 5–12 µm cypovirus polyhedra crystals (Coulibaly *et al.*, 2007 ▸). Fixed-target supports include nylon or polymer loops (Gati *et al.*, 2014 ▸; Hirata *et al.*, 2014 ▸; Gao *et al.*, 2018 ▸), microfabricated polymer mesh (Coulibaly *et al.*, 2007 ▸) or microwell arrays (Guo *et al.*, 2018 ▸), which may be held in a rigid frame (Karpik *et al.*, 2020 ▸), Si and Si/SiN ‘chips’ with through holes (Roedig *et al.*, 2015 ▸; Mueller *et al.*, 2015 ▸; Mehrabi *et al.*, 2020 ▸; Wierman *et al.*, 2019 ▸), a thick polymer sheet with through holes (Baxter *et al.*, 2016 ▸) and a multi-layered structure of polymers (polyimide, polycarbonate, COC or PDMS), silicon and/or silicon nitride (Gicquel *et al.*, 2018 ▸; Shelby *et al.*, 2020 ▸; Feld *et al.*, 2015 ▸; Murray *et al.*, 2015 ▸). Excess liquid can be withdrawn through the holes, and the resulting liquid flows can help to position crystals over/within the holes, reducing the background scatter (Roedig *et al.*, 2015 ▸; Mueller *et al.*, 2015 ▸; Mehrabi *et al.*, 2020 ▸). Deep wells/holes exclude some crystals based on size and morphology (Roedig *et al.*, 2015 ▸; Wierman *et al.*, 2019 ▸). Microfluidic elements can be added to the support for crystal loading and positioning (Lyubimov *et al.*, 2015 ▸; Maeki *et al.*, 2020 ▸), but may retain more liquid around the crystals. Opaque materials such as silicon complicate the optical imaging of crystals. Diffuse scattering from well/hole walls (for example when using amorphous polymers; Baxter *et al.*, 2016 ▸) can restrict oscillation angles and the probing of crystals adjacent to walls. Bragg scattering from crystalline support materials can overload detector pixels. Many fixed-target supports have large thermal masses that compromise cryocooling. Manufacturing costs for Si-based and SiN-based supports can be substantial.

Compared with moving-target approaches, fixed-target approaches typically allow data collection from a larger fraction of the available crystals, with some methods approaching 100% hit rates (Oghbaey *et al.*, 2016 ▸), and at SR sources crystal oscillation during exposure further reduces the number required for a complete data set. Crystals are not size-filtered, are subject to fewer mechanical stresses and may be less likely to be damaged than with moving-target injector methods (Lee *et al.*, 2019 ▸; Martiel *et al.*, 2019 ▸). Fixed-target approaches are thus better suited for many biologically/biomedically important systems for which crystals, and even microcrystals, may not be abundant and crystal morphologies may not be ideal.

Several challenges are important in the design of fixed-target sample supports. Microcrystal visualization should be straightforward to minimize the doses received in blind X-ray raster scanning and data collected with off-beam-center, and thus inhomogenous, crystal irradiation and damage (Warkentin *et al.*, 2017 ▸). Background X-ray scatter from all sources must be minimized over the range of support orientations used in data collection. Crystals must be well dispersed with minimal clustering/overlap (Roedig *et al.*, 2015 ▸; Oghbaey *et al.*, 2016 ▸). A means must be provided to remove excess fluid around crystals without damaging them (see, for example, Oghbaey *et al.*, 2016 ▸) and to maintain crystal hydration during loading, transport to the synchrotron and data collection. For cryocrystallography, the thermal mass of the support must be minimized and the crystals must remain within cryostreams during rastering. For wide adoption, the sample-support system should be compatible with the sample-handling infrastructure at SR sources (Baxter *et al.*, 2016 ▸) and be inexpensive to manufacture and use.

We have developed an integrated fixed-target SSX sample-preparation and handling system, based in part on previously demonstrated concepts, that is easily and inexpensively manufactured, is highly customizable for different crystal morphologies and crystal-handling challenges, has excellent X-ray and optical performance, is suitable for both RT and cryo-SSX, and is compatible with the existing infrastructure for high-throughput mail-in cryocrystallography.

## 2. SSX sample-handling system design   

**Figure 1:**
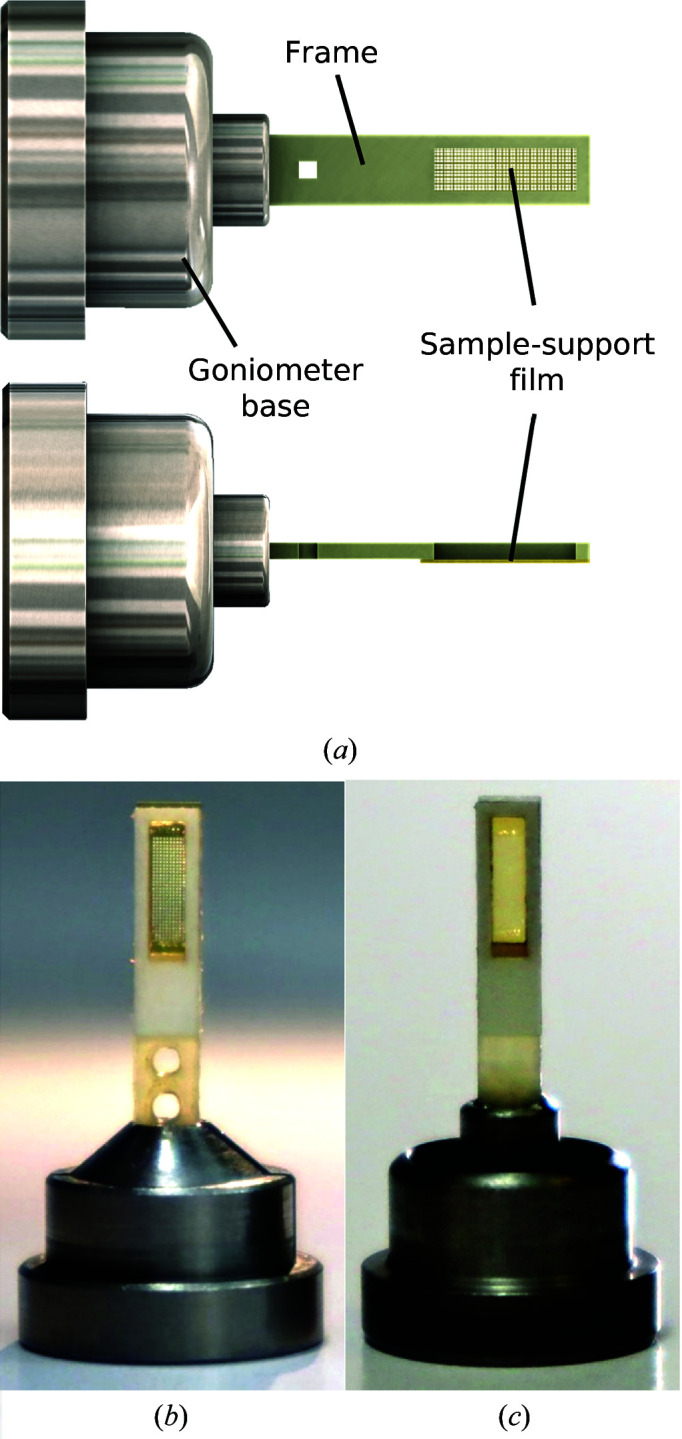
Sample supports for serial synchrotron crystallography (SSX). (*a*) A microfabricated thin film is attached to a thin, rigid frame. For room-temperature data collection, thin sealing films are applied. (*b*, *c*) Front views of sample supports in ALS-style and SPINE-style goniometer bases. The frame shown has a width of 2.5 mm and a 1.5 × 5 mm aperture. The two holes at the bottom end of the frame in (*b*) allow gripping with tweezers when the frame is handled without the goniometer base.

**Figure 2:**
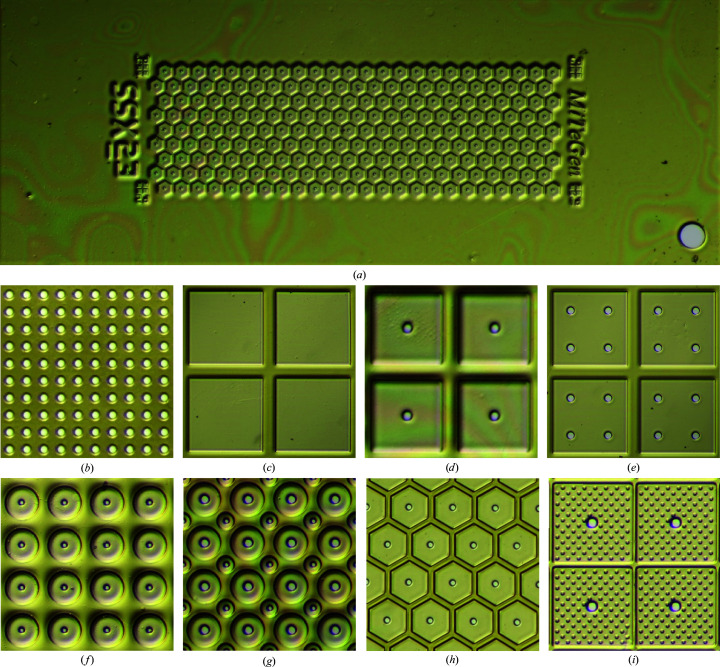
Sample-support films. (*a*) The microfabricated films have an ‘active area’ where crystals and solution are deposited, fiducials to assist in alignment at the X-ray beamline and codes to identify the specific design. The films are 2.5 mm wide and either 3.5 or 6.5 mm long for cryo- or room-temperature data collection, respectively. The film thickness for the prototypes shown is either 4 or 10 µm in the sample windows and 10 or 20 µm elsewhere. Color/contrast is boosted for clarity. (*b*)–(*i*) Examples of sample-support film designs. (*b*) A single well/window covering the entire active area with an array of small holes for liquid removal via back-side suction or blotting (shown: 30 µm holes, 72 µm spacing). (*c*) Square-well array with no holes (shown: 600 µm windows, 100 µm walls). (*d*) Square-well array with one hole per cell (shown: 220 µm windows, 50 µm walls, 20 µm hole). (*e*) Square-well array as in (*c*) with four 50 µm holes. (*f*) Circular wells with a single hole (shown: 100 µm wells, 10 µm holes, 130 µm center to center). (*g*) Circular wells of two sizes (shown: 100 and 50 µm wells, 20 and 10 µm holes, respectively). (*h*) Hexagonal wells (shown: 150 µm center to center, 20 µm walls, 20 µm holes). (*i*) Square wells with an array of posts to immobilize crystals (shown: 220 µm windows, 10 µm walls, 10 µm posts, 20 µm holes).

**Figure 3:**
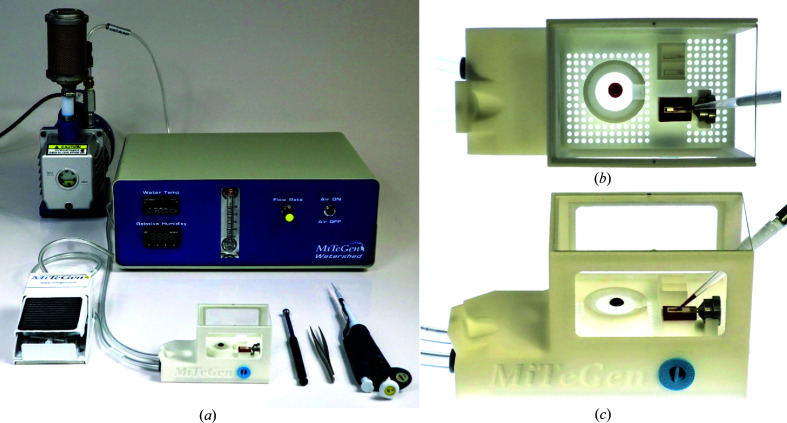
(*a*) Sample-loading system comprised of a station connected to a Watershed humidity-controlled gas-stream generator and to a vacuum pump. A foot pedal allows continuous variation of the suction applied to the station. (*b*) The sample-loading station has receptacles for an SSX sample support (with suction applied beneath the active area of the support via a gasketed port), a glass cover slip containing a crystallization drop and/or buffer, filter paper for back-side blotting and sample-sealing films. Humidity-controlled air flows through holes in the base of the station to maintain sample and drop hydration. The bottom of the station is transparent to allow back-illumination of both the sample-support film and the crystal-containing drop on the cover slip. (*c*) The blue plug is removed when accumulated solution drawn off via suction is to be drained.

**Figure 4:**
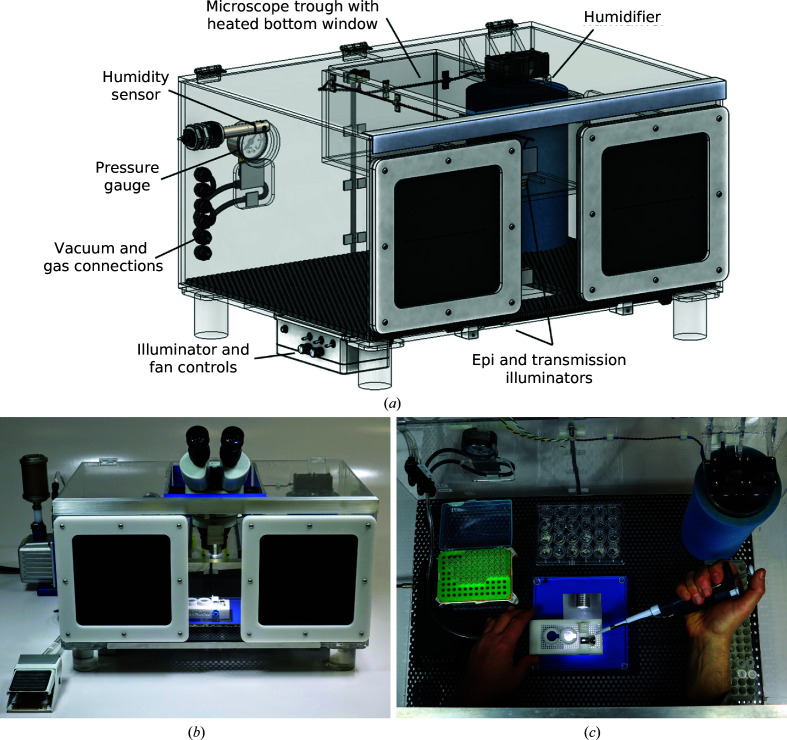
Humidified glovebox to minimize dehydration risks during sample preparation and support loading and sealing. (*a*) Schematic illustration showing the glovebox components, with the stereo microscope and gas and vacuum connections removed. (*b*) Front view of the glovebox. A long working-distance binocular microscope rests on a heated window in a trough in the lid of the glovebox, allowing inspection of the sample-loading station and crystallization trays. (*c*) The glovebox holds the station (here shown with its enclosure removed), crystallization trays, buffer and cryoprotectant solutions, and other tools and supplies needed. Feedthroughs connect the station to vacuum and to a separate humidity-controlled gas stream. The fan-driven humidifier at the upper right raises the humidity within the glovebox from ambient humidity (typically <50% r.h.) to >95% r.h. in roughly 10 min.

Figs. 1 ▸, 2 ▸, 3 ▸ and 4 ▸ show components of our SSX sample-preparation and handling system. Fig. 1 ▸ shows the sample support on a goniometer base. Fig. 2 ▸ shows examples of the films used to support the crystals. Fig. 3 ▸ shows a sample-loading station used when loading crystals onto the support of Fig. 1 ▸. Fig. 4 ▸ shows a humidified glovebox used to prevent the dehydration of crystals and solutions during sample loading.

### 2.1. Sample supports   

As shown in Fig. 1 ▸, sample supports are composed of three components: a microfabricated sample-support film, a rigid frame to which the film is attached and an ALS- or SPINE-style magnetic steel goniometer base modified to capture the frame. The frame size and base style make the supports compatible with all sample-storage and handling devices for high-throughput cryocrystallography, as with the fixed-target supports of Baxter *et al.* (2016 ▸).

#### 2.1.1. Sample-support films   

Thin films to support crystals were microfabricated in polyimide using MiTeGen’s standard processes. These allow the prototyping and production of multiple designs with low up-front design and tooling cost and low incremental production cost.

Polyimide is optically and X-ray transparent, mechanically tough at room and cryogenic temperature, and radiation-hard for a polymer, and is widely used for X-ray sample holders and windows. Mechanical toughness and X-ray damage resistance are essential for sample holders to be reusable. Polyimide has strong optical absorption below ∼380 nm, giving it a gold color. This absorption does not hinder visible-light microcrystal imaging as long as the films are thin (<30 µm); it affects UV excitation fluorescence imaging in transmission but not in epi mode, and it does not affect two-photon excitation fluorescence (TPEF) and second-harmonic generation (SHG) imaging at typical excitation wavelengths near 1064 nm.

The sample-support films had a width and length of either 2.5 × 3.5 mm or 2.5 × 6.5 mm, an active area, where crystals resided, of either ∼1.2 × 2.5 mm or 1.2 × 5 mm, and a base thickness of 10 or 20 µm. Films of this thickness are robust and easy to handle during assembly. The crystal-supporting region of the film was patterned with an array of cells, each with one or more wells/windows where the film thickness was reduced by a factor of 2–3 to ∼4 or 10 µm. Each well typically included one or more through-holes for the removal of excess liquid and crystal repositioning by applying suction to the back side of the film (Mueller *et al.*, 2015 ▸) or blotting. Films included fiducials to define a coordinate system and facilitate alignment in the X-ray beam, and machine-readable text identifying the design (Fig. 2 ▸ *a*).

Crystal sizes and shapes are diverse, and diffraction-quality crystals may be abundant or scarce. Locating crystals at well defined positions on the sample support film allows step-and-repeat data collection, reducing data-acquisition times compared with continuous raster scanning. However, if crystal positions can be reliably recognized by optical means, a random distribution with minimal clustering may give more efficient data collection and crystal use with appropriate scanning algorithms.

No single sample-support film design can optimally address all constraints for all samples. 25 sample-support film designs were fabricated and tested. Representative designs (some of which are shown in Fig. 2 ▸) and their motivations include the following.

(i) A single flat well with no holes or with an array of holes (Fig. 2 ▸ *b*). Thin, uniform-thickness polymer over the entire active area of the support gives excellent imaging and minimizes the X-ray background at all positions. Arrays of square wells, either without (Fig. 2 ▸ *c*) or with (Figs. 2 ▸ *d* and 2 ▸ *e*) holes, were used. The well size (200–600 µm), hole size (20 or 50 µm) and number of holes per well (one or four) was varied. With hole-free designs, liquid could only be removed by blotting from the crystal side of the film, which proved to be much harder to control than liquid removal via suction.
(ii) Arrays of circular wells with a single hole per well (Fig. 2 ▸ *f*). The well size (50, 100 or 200 µm) and hole size (2, 5, 10 or 20 µm) were varied. Circular symmetry might give more uniform liquid removal and crystal streaming to the hole, but the total wall area is increased, so more crystals might be left stranded on the (thicker) walls.
(iii) Arrays of circular wells of two different sizes (Baxter *et al.*, 2016 ▸), with central holes scaled to match. Different well and hole sizes on the same support might increase the chance that some wells have suitably positioned crystals and/or give better results when crystal sizes are heterogeneous. The two well sizes were chosen to minimize the wall area; for the packing of circles, the optimal radius ratio is 0.41:1 (Kennedy, 2006 ▸).
(iv) Hexagonal wells with central holes (Fig. 2 ▸ *i*). A hexagonal array, like a square array, allows walls of uniform narrow width and minimal area and, like a circular well, might give more uniform liquid removal and crystal streaming to holes.
(v) Square (or other shape) wells, each with a central hole and covered by an array of cylindrical posts (Fig. 2 ▸ *d*). The posts might create ‘friction’, impeding crystal flow to the holes and leaving them dispersed in each well after liquid removal (Lyubimov *et al.*, 2015 ▸). Posts and walls help to tilt crystals that partly reside on them out of the main plane of the film and, for crystals of a size at most a few times larger than the wall/post height, should help to reduce preferential crystal orientation.

#### 2.1.2. Sample-support frame   

The sample-support films (Fig. 2 ▸) were bonded using a thermally activated epoxy to a 250 or 500 µm thick frame. The film was attached so that its crystal-holding active area faced into an aperture in the frame. The frame protects crystals from contact with sealing films (Section 2.1.4) and provides additional depth (beyond that of wells patterned into the support film) to retain liquid, which is useful in crystal repositioning and for *in situ* crystallization.

Several frame materials were evaluated, including thin metals and polymers. The fiberglass–epoxy laminate G-10 had the best combination of stiffness (flexural modulus ∼17 GPa), resistance to plastic deformation and damage, and absence of sharp diffraction rings. The thermal expansion coefficient of G-10 (<12 µm m^−1^) is much smaller than that of polyimide (>30 µm m^−1^). Differential contraction of the frame and film on cooling, following thermal curing of the epoxy and during cryocooling, then leaves the film in tension. The film remains flat rather than buckling, which is important in imaging, large-rotation data collection and data processing.

#### 2.1.3. Goniometer base   

The sample-support film plus frame was inserted into a magnetic steel goniometer base modified to capture the frame. Prototypes (Fig. 1 ▸ *b*) used a commercially available ALS-style base with a set screw (Crystal Positioning Systems CP-111-070). The dimensions of the frame were chosen so that any beamline-compatible goniometer base could be modified to accept them. Current designs (Fig. 1 ▸ *c*) use modified ALS- and SPINE-style bases that allow the easy separation of frames and bases for storage, *in situ* crystallization, cleaning, reuse and recycling.

#### 2.1.4. Sealing films and room-temperature storage   

For room-temperature data collection, the sample support must be sealed to minimize dehydration prior to and during X-ray data collection. The experiments in Section 4.3 led to the use of a 3.2 µm thick Mylar sealing film which was attached to the frame using a thin laser-cut double-sided adhesive gasket.

For room-temperature storage for hours to days (sufficient for transport to a synchrotron as in the present experiments at NSLS-II), the sealed sample film plus frame plus goniometer base was placed in a modified magnetic cryovial containing an absorbent polymer plug soaked with, for example, reservoir solution or crystallization buffer. For longer term storage, commercial *in situ* crystallization and storage trays accepting goniometer-base-mounted sample supports, based on the designs of Baxter *et al.* (2016 ▸), can be used. Alternatively, the sealed sample film plus frame can be removed from the goniometer base and stored in, for example, an Eppendorf tube containing a solution-soaked absorbent polymer plug or in a custom-designed *in situ* crystallization and storage tray.

#### 2.1.5. Sample-support dimensions   

The sample-support dimensions, given in Supplementary Table S1, were chosen for compatibility with existing hardware for home-laboratory handling, storage and shipping, and automated data collection at synchrotrons (Baxter *et al.*, 2016 ▸). The most severe constraints are imposed by the inside diameter of the automounter grippers (∼3 mm), which were used to transfer samples from pucks/cassettes into the X-ray beam, by the internal height of UniPucks, by the ∼1 cm diameter of cold nitrogen-gas cryostreams, which are typically directed off-axis, and by the limited *xy* ranges for fast rastering. These constraints dictated the frame width and length, the support film width and the overall length of the assembly.

Although sample supports with much larger active areas have been demonstrated, the larger areas provide little benefit for data collection at synchrotrons. Firstly, the areas of our supports (and our frame thickness) are more than sufficient to contain all crystals (and all liquid) from typical crystallization drops with volumes of <2 µl, which will be used to produce the vast majority of SSX samples. Secondly, data-acquisition times from our supports using bright synchrotron beams will generally be much larger than sample-exchange times. The dose rate 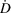 (in MGy s^−1^) delivered by an X-ray beam with uniform flux *F* (in units of 10^12^ photons s^−1^) in an area *A*_beam_ (in µm^2^) is roughly 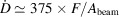 for crystals lacking significant absorption from heavy atoms such as lysozyme (Zeldin *et al.*, 2013 ▸; Warkentin *et al.*, 2017 ▸). For an exposure equal to half the half-dose *D*_1/2_ (in MGy) at positions separated by the (circular) beam diameter over the area *A* of a sample support, the total exposure time Δ*t*, which excludes the time for initial alignment, translations without exposure and oscillation overhead (if samples are oscillated at each point) *etc.*, is then 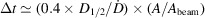. With *A* in mm^2^ and Δ*t* in minutes, this becomes Δ*t* = 18 × *D*_1/2_ × *A*/*F*. Using a typical room-temperature half dose of ∼0.2 MGy for ∼1.5 Å resolution data (Warkentin *et al.*, 2017 ▸) and a flux *F* of 1 × 10^12^ photons s^−1^ (for example for the FMX beamline at NSLS-II), the exposure time per mm^2^ of sample support is then ∼3.6 min. For the 1.2 × 2.5 and 1.2 × 5 mm active areas of our sample-support films, the exposure times would then be 11 and 22 min, respectively, compared with sample-exchange times of well under 1 min. Using a cryogenic temperature half dose of ∼15 MGy for ∼1.5 Å resolution data (Teng & Moffat, 2000 ▸; Atakisi *et al.*, 2019 ▸), these exposure times increase to 825 and 1650 min (13.8 and 27.5 h), respectively. These values, which assume that the entire support area is scanned with the same (maximum) exposure per unit area, represent upper bounds that might be approached if the support is densely covered with microcrystals and, for example, only rare crystals of a particular polymorph yielded adequate diffraction. Under more typical coverage conditions, data-acquisition times might be smaller by an order of magnitude at room temperature and by a larger factor at cryogenic temperatures. Even so, in most applications, the costs of constraining the size of the sample support as we have done will be modest compared with the benefits of maximum compatibility with the existing infrastructure.

### 2.2. Sample-loading system   

Fig. 3 ▸ shows the sample-loading system, which includes elements of previous sample-loading systems (Oghbaey *et al.*, 2016 ▸; Mehrabi *et al.*, 2020 ▸). The system allows crystals and mother liquor/buffer/cryoprotectant solutions to be dispensed onto sample supports in one or multiple steps and the removal of excess liquid by suction or blotting, all within a controlled-humidity environment to minimize dehydration and maximize crystal isomorphism.

Fig. 3 ▸(*a*) shows the sample-loading station. The station holds the sample support, a cover slip with drops of crystal-containing solution, buffer and/or cryoprotectant solution, sealing films and filter/blotting paper. It has connections for vacuum and humidified air. A vacuum port with a sealing gasket lies directly under the sample-support film. Humidified air flows upward through the hole array covering the work surface of the station. Optically clear windows at the sides and top allow visualization by eye or using a microscope. The optically clear bottom allows backlighting of the cover slip and sample-support film for optimal imaging.

Vacuum is provided by a small vacuum pump or by a Bernoulli-principle compressed-air vacuum generator. The gauge pressure applied at the vacuum port is continuously variable from 0 to ∼0.2 bar (20 kPa) using a custom-built foot-pedal control, leaving both hands free for other manipulations. The time profile of the applied vacuum and thus the rate of liquid removal during sample loading can be optimized for different film designs, crystal sizes and solution viscosities.

Air with controlled humidity up to ∼100% r.h. is provided by MiTeGen’s Watershed humidified air generator, based on an earlier design (Farley *et al.*, 2014 ▸).

### 2.3. Humidified glovebox   

As an additional defense against crystal and crystallization-drop dehydration, the sample-loading station, crystallization plates, buffers and other required tools and materials are housed within a humidified ‘gloveless’ glovebox that includes an integral microscope imaging system, as shown in Fig. 4 ▸. Wierman *et al.* (2019 ▸) used a humidified enclosure with their vacuum ‘chuck’ when loading their silicon-well SSX chips. Unlike previous humidified enclosures used in crystallography and commercial humidified gloveboxes, our design provides, and allows the measurement of, the near-saturating humidities (>95% r.h.) that correspond to typical water activities in protein crystals, which is critical to preventing dehydration of the smallest crystals and for maximizing working times with crystallization trays.

The glovebox has generous dimensions of roughly 60 × 40 × 30 cm. Interior access is provided through a hinged top, a side door and two hand ports. The hand ports each have two natural rubber diaphragms with slits oriented 90° from each other. This *XY* slit arrangement tightly seals around a user’s hands/arms as they are inserted, allowing internal humidity levels near 100% r.h. to be maintained without the use of cumbersome attached-glove entry ports.

Several methods were explored to rapidly generate and then maintain near-saturating humidities while minimizing fogging and condensation on glovebox surfaces. In the glovebox prototype shown in Fig. 4 ▸, humidity is generated using fans that blow air through a water-soaked absorbent foam ‘sock’, which is easily removed for cleaning. This system brings the interior humidity above 95% r.h. without heating in ∼10 min and minimizes fogging and condensation. A humidity sensor designed for use in near-saturating humidities records the internal humidity with better than 1% r.h. accuracy.

The glovebox in Fig. 4 ▸ has several other features that are optimized for SSX sample handling and loading. A long-working-distance binocular microscope mounted on a custom sliding stand rests in a transparent glass- or acrylic-bottomed trough in the lid of the glovebox for imaging of crystallization trays, cover slips and sample supports. Heaters on the trough bottom prevent fogging. High-contrast sample illumination is provided by a custom transmission illumination system mounted on the bottom of the glovebox and by a custom epi illuminator mounted in the trough; the trough also has room to attach a standard ring illuminator to the microscope pod. The microscope, illuminators and all wiring are located outside the humidified volume to prevent fogging and long-term corrosion/degradation by saturating humidities. The glovebox has connections for vacuum/suction, for two-stage humidity control using an exterior Watershed humidity generator connected to the sample-loading station and for flushing with, for example, dry air or N_2_ gas, as well as a pressure-release valve.

The sample-loading station and glovebox described here have some similarities to the system recently described by Mehrabi *et al.* (2020 ▸). The key advantages here are the greater flexibility of our sample-loading station, easier sample visualization and sealing, convenient variable control of vacuum/suction via the foot pedal, and much higher (>97% versus 85%) and much better (two-stage) control of humidity, which is critical to maintaining crystal hydration and isomorphism and maximizing working times.

## 3. SSX system evaluation and evolution   

### 3.1. Protein crystals   

Three proteins, fluoroacetate dehalogenase (FAcD), hen egg-white lysozyme (HEWL) and human glutaminase C [both the apo form (apo hGAC) and with a bound inhibitor (hGAC-I)], were used to evaluate our system. The FAcD crystals had dimensions of 10 × 10 × 40 to 20 × 20 × 60 µm. Three crystal forms of lysozyme, tetragonal, orthorhombic and monoclinic, were prepared. Tetragonal crystals ranged from 40 × 40 × 40 to 2 × 2 × 2 µm, while orthorhombic and monoclinic crystals had dimensions of ∼50 × 50 × 50 µm and ∼30 × 30 × 120 µm, respectively. The cystals of apo hGAC and hGAC-I were much larger, averaging 100 × 100 × 200 µm. Crystallization details are given in Supplementary Section S1.

### 3.2. Sample loading   

The sample-loading station and all supplies and tools are placed in the humidified glovebox, which provides a humidity >97% r.h. (with 0.8% r.h. uncertainty). A sample support is placed in the station, and the station humidity was increased to >98% r.h. (or to the expected r.h. of the crystals if lower). To verify proper seating of the sample support on the vacuum port, reservoir solution is deposited on the support and vacuum is applied to remove it. The sample supports are then loaded with 5–10 µl crystal-containing solution. Crystals may be allowed to sediment onto the support film, or else appropriate suction can be applied immediately (for example after crystal and liquid deposition, after additional liquid is added and aspirated or after the support has been inverted to allow sedimentation to the air–liquid interface) to draw suspended crystals and liquid towards holes in the support. Additional liquid, with or without crystals, can be dispensed to obtain a desired crystal density. After the final removal of excess liquid, liquid that may have transferred to the back side of the support can be blotted using strips of filter paper. In some experiments, only back-side blotting (no vacuum) was used to remove excess liquid surrounding crystals. For room-temperature data collection, sealing films were then applied.

FAcD crystals were also prepared for cryogenic data collection. Crystals grown in 2.5 µl drops on cover slips were resuspended using 10 µl well solution mixed with 10 µl of a 40% glycerol solution. 15 µl of this crystal solution was then loaded onto the sample support and excess liquid was removed using vacuum and back-side blotting as at room temperature. Samples were then immediately plunge-cooled using MiTeGen’s Nanuq automated hyperquenching cryocooler and automatically stored in UniPucks, which were then transferred to storage dewars.

### 3.3. X-ray data collection and evaluation   

#### 3.3.1. Data collection and processing   

Data from FAcD crystals were first collected at *T* = 100 K on the XF17ID2 (FMX) beamline at NSLS-II and then at room temperature on the ID7B2 (FlexX) beamline at CHESS. Data from lysozyme and hGAC crystals were collected at room temperature on ID7B2. Beamline experimental parameters are given in Supplementary Section S2.

SSX sample supports were mounted as ordinary crystallo­graphy samples on a goniometer head attached to an air-bearing goniometer. In experiments on FMX using FAcD samples cooled to 100 K, the sample support was rastered relative to the beam in steps of 20 µm, and 5° of oscillation data were collected at each position. In experiments on ID7B2 using room-temperature FAcD and lysozyme samples, crystals were point-and-click selected and 5° of oscillation data were collected. In experiments using hGAC, the support was rastered in 20 µm steps and 5° of oscillation data were collected. Each step in the raster could be completed in 0.75–0.5 s for stepping and 0.25 s for data acquisition (25 frames, 0.2° and 10 ms per frame), corresponding to a 1.3 Hz raster rate. This rate, which was achieved using an air-bearing goniometer for oscillations, compares with a 3 Hz rate achieved by Wierman *et al.* (2019 ▸) using a dedicated oscillation stage.

Individual oscillation-frame sets were processed with *XDS* and scaled and merged together with *XSCALE* (Kabsch, 2010 ▸). The detailed processing and filtering routine using *XSCALE_ISOCLUSTER* (Brehm & Diederichs, 2014 ▸) has been described previously (Wierman *et al.*, 2019 ▸). Phasing and molecular replacement were performed using *Phaser* and *phenix.refine*, respectively, in *Phenix* (Liebschner *et al.*, 2019 ▸).

## 4. Results   

### 4.1. Sample-support use and performance   

The modular sample-support configuration and an efficient pipeline for the microfabrication of sample-support films facilitated cycles of design, testing and optimization, and yielded insights into how sample supports can best be used with diverse crystals.

#### 4.1.1. Key principles in sample loading   

Optimal sample-support film designs and loading procedures depend on the crystal size and shape and on the solution viscosity. These determine the sedimentation speed of the crystal, and thus how quickly it settles onto the support film. Sedimentation speed varies with crystal size *L* and viscosity η as *L* ^2^/η, and in water is ∼500 µm s^−1^ for *L* = 50 µm, ∼20 µm s^−1^ for *L* = 10 µm and <1 µm s^−1^ for *L* = 2 µm. Larger crystals will sediment onto the sample-support film in <1 s, whereas smaller crystals may remain suspended above the film for ∼1 min or more.

Liquid flows caused by suction or blotting will generally be laminar. The flow velocity must be zero adjacent to the sample-support film and increase with height above it. Crystals that have sedimented into contact with a flat film will be acted on by a friction force proportional to their apparent weight in the liquid (∝*L* ^3^) and by a viscous force that depends on their projection above the film (∝*L*), the fraction of the far-field flow velocity that has been achieved at that height (∝*L* for small *L*) and on the overall flow rate. For sufficiently small flow rates, static friction with the film will win and the crystal will remain where it landed. The threshold flow rate will roughly be proportional to *L*, so larger crystals will be harder to dislodge than small ones. Crystals that have not sedimented into contact with the film will flow with the liquid at a speed at most equal to that of the fluid immediately surrounding them, which will depend on their height above the film.

The goal in sample loading may be to concentrate crystals at regularly placed holes in the support film in order to reduce the area that must be scanned by the X-ray beam during data acquisition (for example, when crystal positions may otherwise be hard to determine). After the solution and crystals have been deposited, suction applied immediately to the back side of the film will cause the many crystals that remain suspended to flow with solution toward the holes. Once the crystals have sedimented into contact with the film, the film can be inverted, allowing the crystals to sediment to the air–liquid interface, then flipped back and suction applied immediately to draw solution and crystals to the holes. Crystals can also be resuspended by adding liquid and aspirating. Larger crystals sediment much faster, so much more liquid is required to increase the average distance (and time) that the crystals must sediment before they contact the support film. Suspending crystals larger than ∼30 µm for long enough for them to flow to holes before contacting the film is impractical, and so large flow rates are required to overcome friction with the support if positioning large crystals at holes is the objective.

Once crystals have flowed to a hole, they may partially block it, restricting flow, and cause crystals to stream toward another open hole. If too much suction is applied, the liquid film above a hole may break, allowing air to flow through. This reduces liquid and crystal flow to that hole and also (to a lesser extent) to other holes that are still liquid-covered. To minimize crystal clustering, the number of crystals loaded onto the support should preferably be comparable to or less than the number of holes.

Dividing the support surface into wells using walls reduces clustering by reducing the area from which crystals can be drawn to a given hole. ‘Short’ walls (as in the current designs) are effective in reducing crystal motion if crystals have sedimented to near or below the wall height, or if the top of the solution layer has been lowered to within a factor of ∼2 of the wall height. Filling the entire active area of the current designs to twice the wall height requires ∼100–400 nl of crystal-containing solution. When larger solution volumes are dispensed, weak suction can initially be applied to lower the liquid level toward the wall height, when the liquid surface will develop a visible undulation matching the wall pattern. Strong suction can then be applied to draw crystals toward the holes in each well.

The goal in sample loading may instead be to disperse the crystals randomly but uniformly on the sample support to minimize crystal clustering and diffraction overlap. This generally allows far more crystals per unit area to be loaded than when the goal is one crystal per hole. Crystals should be given ample time to sediment onto the supporting film, and solution removed by applying weak suction. Posts and similar features within wells project up into the solution, reducing liquid velocity and impeding crystal flow and clustering when suction is applied. Final crystal positions can be determined using one or more of several common optical imaging modalities [for example visible, UV fluorescence (UVF), second-harmonic generation (SHG; Kissick *et al.*, 2011 ▸) and two-photon excitation fluorescence (TPEF; Padayatti *et al.*, 2012 ▸)]. These data can be analyzed to determine an optimal rastering strategy.

The size of the holes in the support film determines both the minimum crystal size that can be trapped at the holes (without passing through) and the liquid flow rate. For holes in the size range of relevance, experiments on water and water–glycerol mixtures show that the flow velocity depends mainly on the pressure difference Δ*p* across the film and is nearly independent of the hole diameter and solution viscosity, in contrast to Poiseuille flow through tubes (Hasegawa *et al.*, 2015 ▸). Consequently, the volume flow rate through the hole 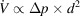, where *d* is the hole diameter. For a pressure difference of 10 kPa (0.1 bar) and water with viscosity of 1 mPa s (or a water–glycerol mixture with a viscosity of 0.1 mPa s), the measured flow rates through 50, 20 and 10 µm holes are ∼4.5, 0.63 and 0.13 µl s^−1^, respectively (Hasegawa *et al.*, 2015 ▸). Hole diameter thus has a strong influence on flow-induced crystal positioning. Hole diameter also determines the minimum pressure difference Δ*p* required to overcome surface tension and drive liquid through the hole, according to Δ*p* ≃ 4γ/*d*. For 1 and 10 µm diameter holes, Δ*p* is 290 and 29 kPa, respectively, corresponding to ∼3 and 0.3 bar.

#### 4.1.2. Observed sample-loading performance   

**Figure 5:**
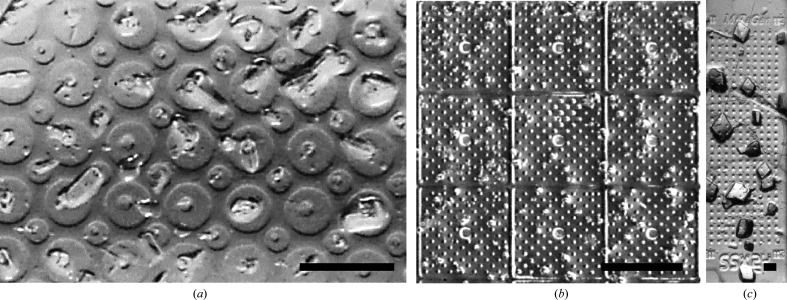
Crystals of (*a*) FAcD, (*b*) tetragonal lysozyme and (*c*) hGAC-I on SSX sample supports after liquid removal. The scale bars are 200 µm in length.

The observed performance of our sample-support film designs was generally consistent with the ideas and principles described in Section 4.1.1. Examples of crystals on supports are shown in Fig. 5 ▸.

Continuously adjustable suction via the foot pedal provided far more control over liquid removal and crystal positioning than either on–off suction control or blotting with filter paper. Blotting from the crystal-containing side of the support caused uncontrolled liquid and crystal flows and loss of crystals. Blotting from the back side only drew liquid through holes larger than 10 µm, and again in a largely uncontrolled manner. Back-side blotting was useful when liquid remained on the back side, away from the holes, after top-side liquid removal by suction.

The design in Fig. 2 ▸(*b*), which had a single large well covered with an array of holes and no ‘walls’, worked well for larger crystals that had sedimented onto the support, when the goal was liquid removal but not crystal repositioning.

Designs with large (200–600 µm) wells having a single hole of 20 µm in diameter allowed good crystal positioning at holes with minimal clustering (when the loaded crystal density was suitably adjusted) for ∼20–40 µm crystals; these obstruct a hole, reducing further solution and crystal flow to the hole. Smaller crystals provided less obstruction and clustering at holes was more likely, especially if large suction was immediately applied or if loaded crystal densities were high. Larger crystals sedimented onto the support film and tended to stay there during suctioning. For these large crystals, step-and-repeat data collection at regularly spaced locations has no significant advantage, and point and click (or automated recognition and translation) combined with local rastering to paint the full area of the crystal with X-rays is more efficient.

Designs with small (100 or 50 µm) circular or hexagonal wells and 10 or 20 µm holes gave excellent performance with smaller (10–40 µm) crystals of all tested morphologies, allowing crystal positioning at holes and higher loading densities without clustering. Hexagonal wells left fewer crystals ‘stranded’ on the top of walls between wells after liquid removal and thus reduced the polyimide X-ray background for these crystals. Support films with two different size wells were useful when crystal sizes were heterogeneous.

Liquid removal via suction became ineffective when holes were reduced to the 1–2 µm needed to trap (rather than pass) microcrystals of that size. For these crystals, achieving reliable positioning at specific locations without clustering is hard, and the required dividing walls reduce the diffracting crystal volume per unit area of the support. However, very small crystals are more likely to be abundant, and raster scanning the entire support area (or a subset of its area where crystal densities are adequately high) can be efficient provided that crystal clustering and overlap in the X-ray beam are minimized or can be accounted for in indexing. For these small crystals, designs in which the well surface was decorated with an array of posts and where the hole size was 10–20 µm (Fig. 2 ▸ *i*) were most effective. The posts (where the flow velocity must go to zero) ‘capture’ or impede the flow of microcrystals, so crystals tended to remain widely dispersed with sufficiently gentle suctioning.

The optimal amount of suction applied during loading varied with crystal size, sample support-film design and whether the goal was to concentrate crystals at holes or leave them widely dispersed. To concentrate larger crystals (>20 µm) that sedimented rapidly, pipetting ample excess liquid on the support and applying strong suction immediately after liquid plus crystal loading was effective in creating the liquid flows needed to move crystals towards holes. To leave smaller crystals dispersed, allowing them ample time to sediment onto the support film and then applying weak suction to remove liquid worked best. For both large and small crystals, some residual liquid should be left on the support to maintain hydration if diffraction data are to be collected at room temperature. This was achieved by tapering off suction once most crystals had reached their final positions. Suction was applied when removing liquid and repositioning crystals for up to ∼10 s.

The optimal amount of suction also depended on the liquid viscosity. As discussed in Section 4.1.1, the flow rate through the hole itself, for a given pressure difference, does not vary with viscosity. However, the flow rate away from the hole, across the surface of the support, decreases with increasing viscosity. With higher viscosity (for example PEG-containing) solutions, solution above a hole may be removed before solution can flow across the surface of the support film to replace it, dimpling and then breaking the liquid film and allowing air to flow through the hole. Once this occurs, liquid flow through the hole abruptly drops. Using less suction can thus give more efficient liquid removal and crystal repositioning when solutions are more viscous.

### 4.2. Imaging crystals on sample supports   

Imaging crystals on sample supports in the home laboratory (Fig. 5 ▸) and on the beamline (Supplementary Fig. S1) was straightforward, even for crystals as small as 2 µm. Thin polyimide in the wells gave a uniform, weakly tinted background, with less contrast/refraction at the holes than in standard microfabricated crystallography loops. Imaging was cleanest when the crystals resided in hole-free regions of the window of a cell.

Because our system allows efficient liquid removal from around the crystals, the refractive-index difference determining reflection and refraction at the crystal surface is effectively the refractive index of the crystal minus that of air, or roughly *n*_cryst_ − *n*_air_ ≃ 1.55 − 1 = 0.55. This is much larger than the index difference *n*_cryst_ − *n*_liquid_ ≃ 1.55 − 1.33 = 0.22 when crystals are surrounded by liquid. Crystal contrast is thus much greater than with loops or SSX holders when liquid is not removed, as illustrated in Figs. 5 ▸(*a*) and 5 ▸(*c*) and Supplementary Fig. S1. Supplementary Section S3 discusses imaging results obtained SHG and TPEF, which allowed easy detection of 2 µm crystals on the supports (Supplementary Fig. S2).

### 4.3. *In situ* crystallization   

**Figure 6:**
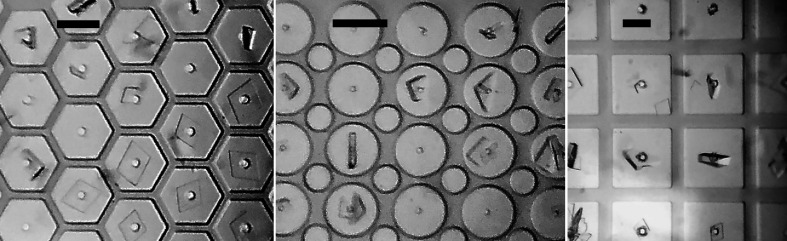
hGAC-I crystals grown by vapor diffusion *in situ* on sample supports suspended above reservoir solution. A large majority of the crystals nucleated directly above (or within) the through-holes in the support film, mostly likely due to additional evaporation and slightly larger supersaturation there. The scale bars are 100 µm in length.

Fig. 6 ▸ shows examples of hGaC-I crystals directly grown on sample supports using vapor diffusion. Crystal growth on conventional and serial crystallography sample supports of all kinds has previously been performed, as well as using gonio­meter-base-mounted crystallization devices (Broecker *et al.*, 2018 ▸). In the present case, the crystals had a strong, and extremely useful, tendency to nucleate and grow directly above the holes. Other steps/edges in the polyimide film, for example at walls between cells, did not promote nucleation, suggesting that hole topography was not a factor, and no such clear preferential nucleation has previously been reported for sample supports that have densely spaced arrays of holes (see, for example, Lieske *et al.*, 2019 ▸). Evaporation through holes should locally increase protein supersaturation there. Crystal nucleation rates increase extremely rapidly with supersaturation beyond a threshold supersaturation. Even a tiny local increase in supersaturation could trigger nucleation at holes, with subsequent protein depletion by growth of that nucleus suppressing nucleation elsewhere. *In situ* crystallization by vapor diffusion on films with a small area fraction of holes may prove to be a highly effective approach to achieving regularly positioned crystals.

### 4.4. Retention of crystal hydration   

Maintaining the hydration, and thus the isomorphism, of all crystals to the end of data collection is critical to maximizing the data quality and minimizing the sample volume required for structure determination. This becomes more difficult as crystal sizes decrease towards 1 µm and when most of the surrounding solution is removed to minimize background scatter. Exposure to room air for seconds can significantly dehydrate microcrystals.

Initially, sample-support loading was performed using the humidified sample-loading station placed in ambient air. Crystal dehydration (as indicated by the unit-cell parameters) occurred if the sample support was not properly seated on the vacuum port of the station, causing ambient air to be drawn into the station when suction was applied, and if the sample support was removed from the station during adjustment and sealing of the Mylar films. To remedy these problems, the vacuum-port sealing gasket was improved, humidified air vents between the vacuum port and the open end of the station were added, and the sealing-film system and protocol was improved. These did not prove to be sufficient to prevent dehydration due to common user errors, so the humidified glovebox was constructed to enclose the station and all supplies and tools. Even without separate and more precise humidification of the station, the 98–100% humidity environment of the glovebox proved to be adequate in most cases (Section 4.6), allowing the top and sides of the station to be removed to simplify access during sample loading.

Dehydration of samples by vapor transmission through sealing films and gaskets in ambient air and by moisture absorption by the G-10 frame was evaluated by filling the aperture of the sample support with a saturated NaCl solution (solubility of 357 mg ml^−1^ at 25°C) and sealing. As water was lost, a salt crystal would eventually nucleate and grow. The time at which this single nucleated crystal reached a size of 50 µm, corresponding to the loss of ∼0.75 nl of water, was found to be ∼20 min. The volume of residual liquid on the sample-support film after suction was between ∼25 and 250 nl, corresponding to solution film thicknesses of between ∼2 and ∼20 µm. A 3% liquid loss then corresponds to a working time between 20 and 200 min. To increase this working time without degrading X-ray performance, the frame of the sample support has been modified to include a second aperture vapor-connected to the first that holds an additional 600–800 nl of solution.

### 4.5. Background X-ray scattering and data-collection/oscillation angular range   

**Figure 7:**
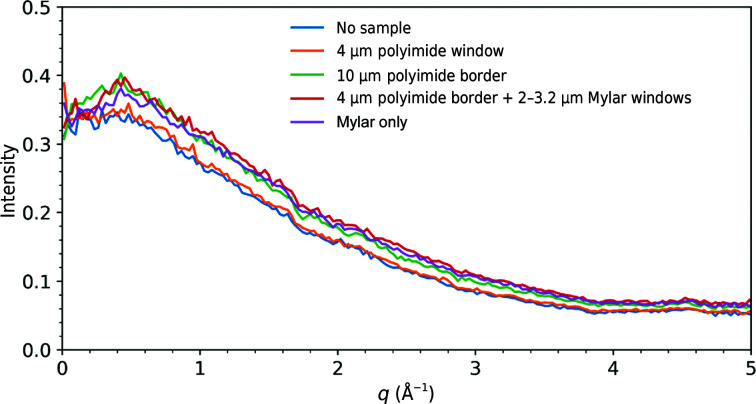
Azimuthally integrated diffraction intensity versus *q* recorded on the FMX beamline at NSLS-II with no sample support, and with the beam passing through a polyimide cell window, the thick polyimide border around the windows and through a polyimide window and two Mylar sealing films. The 1 cm air path of the beamline is calculated to give scattering equivalent to 8 µm of polyimide. Mylar films are not needed for *T* = 100 K data collection.

Fig. 7 ▸ shows measurements performed on the FMX beamline at NSLS-II of the azimuthally integrated diffraction intensity versus *q* measured when the beam passes through a ∼4 µm thick polyimide cell window, a window and two Mylar sealing films, an ∼10 µm thick wall and when the sample support is removed and scatter is generated only by the ∼1 cm air path of the beamline and other beamline components. The additional scatter from the window, walls and sealing films are modest and have only gradual azimuthal variation, facilitating accurate background subtraction.

The G-10 frames produce only diffuse scatter, so detector overload if the direct beam hits the frame is less likely than for silicon-based supports. With a frame thickness of 500 µm and the sample-support film on the detector side of the frame, the diffracted angle range is nearly the full ±90° maximum range and the maximum rotation-angle range (limited by the incident beam hitting the frame) is ∼143° (±71.6°) for cells in the center of the support film and ∼105° (+78.7°, −26.6°) for cells closest to the edge of the support film. These ranges are likely to be sufficient for most structure determinations and allow support rotation to improve reciprocal-space coverage if the crystals are preferentially oriented.

### 4.6. X-ray data processing and refinement   

**Figure 8:**
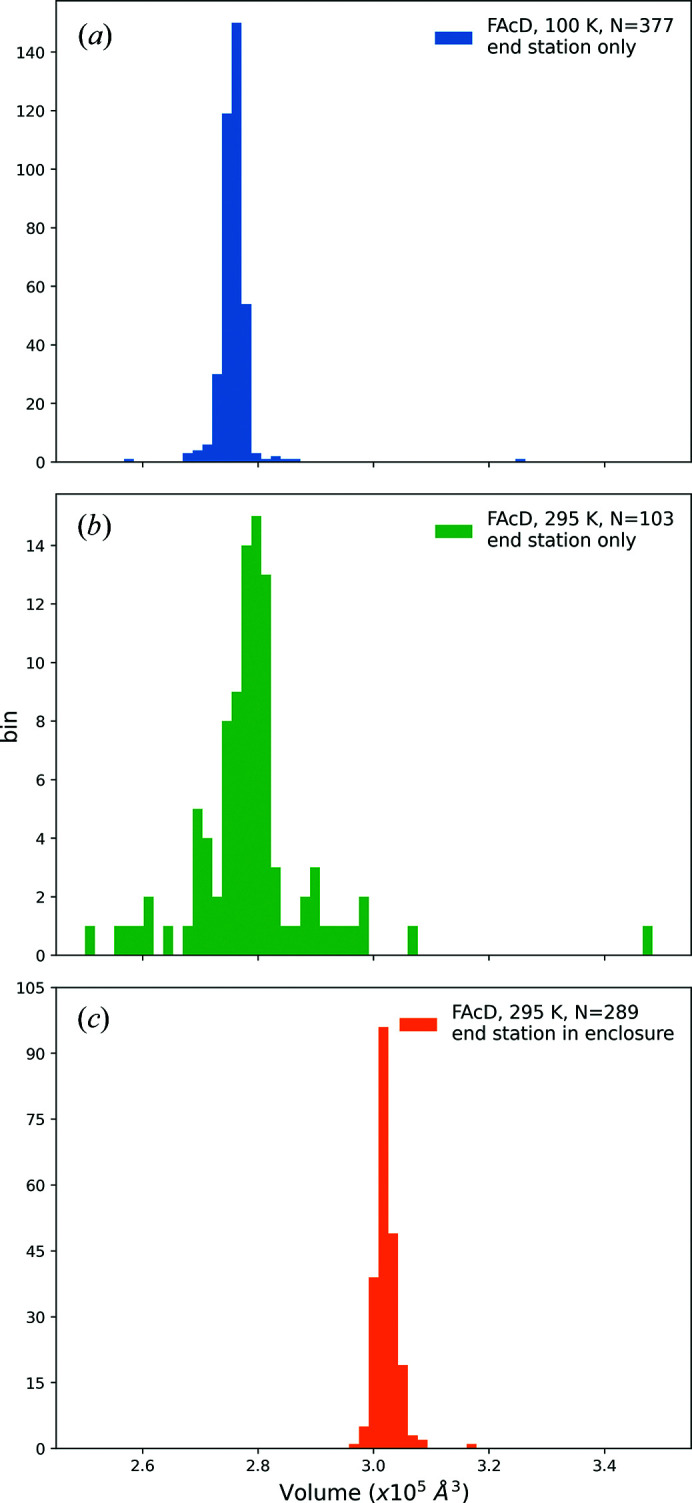
Distribution of unit-cell volumes determined from FAcD crystals using *XDS*. (*a*) Data collected at 100 K from 377 crystals on a single support and (*b*) at room temperature from 103 crystals on a single support, without use of the humidified glovebox. (*c*) Data collected at room temperature from 289 crystals on a single support (chip 6 in Supplementary Fig. S3), prepared using the humidified glovebox.

Fig. 8 ▸ shows unit-cell volume distributions for FAcD crystals at 100 K (Fig. 8 ▸ *a*) and room temperature (Fig. 8 ▸ *b*) before the humidified glovebox and other improvements described in Section 4.4 were implemented, and at room temperature after these were implemented (Fig. 8 ▸ *c*), in each case for crystals on a single sample support. The pre-glovebox room-temperature data show a large unit-cell volume distribution and a smaller than expected mean cell volume, indicating dehydration. The pre-glovebox 100 K cell distribution in (*a*) is narrow, suggesting that the dehydration in (*b*) occurred mainly when the sealing films needed for room-temperature data collection were being applied. The room-temperature unit-cell volume distribution in Fig. 8 ▸(*c*), obtained using the end station within the humidified glovebox, is narrow. Inspection of unit-cell volumes versus the order in which the crystals were measured (Supplementary Figs. S3 and S4) shows that in cases where the sample supports were improperly sealed or became unsealed during data acquisition, unit-cell volumes decrease over time, indicating dehydration. Otherwise, no evidence of dehydration through the sealing films during data collection was observed. The data in Figs. 8 ▸(*a*) and 8 ▸(*b*) were acquired at FMX, while the data in Fig. 8 ▸(*c*) were acquired at CHESS.

**Figure 9:**
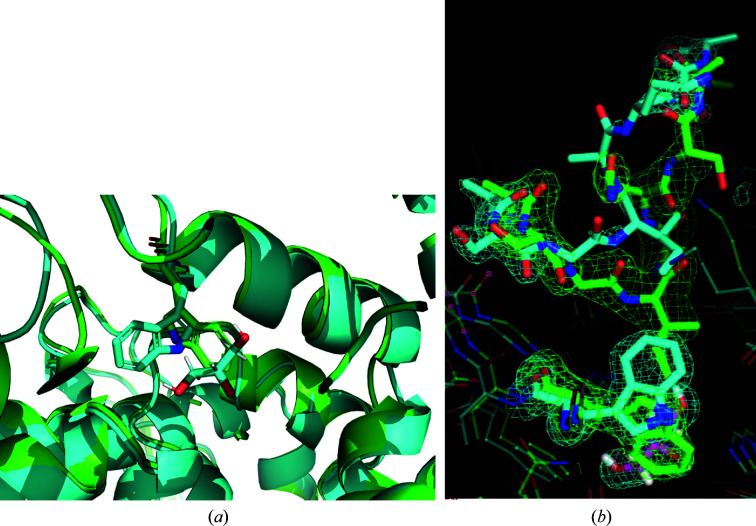
Disordering of the *B*250–*B*258 loop in FAcD with the incorporation of glycerol at low temperature. The room-temperature structure is in green and the 100 K structure is in cyan. (*a*) The side chain of Trp185 is flipped when glycerol is incorporated. (*b*) Disorder of the *B*250–*B*258 loop.

As indicated in Supplementary Table S2, a complete FAcD data set collected at 100 K from 377 crystals on a single sample support was processed smoothly with good statistics. Data from 119 crystals were rejected in processing. Diffraction patterns were free of ice and excess diffuse scatter. The 100 K unit cell was consistent with previous measurements, the mosaicity and Wilson *B* factors were both low, and the CC_1/2_ values were very close to 1. The refined *T* = 100 K structure of FAcD revealed a major modification associated with the glycerol cryoprotectant. Glycerol is a popular small-molecule cryoprotectant, but its small size facilitates binding to proteins, including in active sites. Fig. 9 ▸ shows that glycerol has been incorporated into the active site of FAcD at 100 K, and that this incorporation flips the Trp185 side chain, which in turn disorders the loop between residues 250 and 258.

Data collected at room temperature from 897 FAcD crystals on six sample supports, prepared using the glovebox, also processed smoothly. Twinned crystals displaying pseudo-orthorhombic patterns (252 of the 897) were removed from further processing. After merging and filtering datasets using *XSCALE_ISOCLUSTER*, the best 166 datasets (which had a correlation strength greater than 0.7) were scaled and merged together to generate a complete dataset for structure solution and refinement. This dataset yielded very good statistics, given in Supplementary Table S2. Preferential crystal orientation was not a significant issue (Supplementary Fig. S5).

Supplementary Table S2 also gives statistics for a complete orthorhombic lysozyme data set collected at room temperature. In this case the unit-cell distribution was narrow (SD of ∼0.6%) and no evidence of dehydration was observed, even though the original sample-loading station without the glovebox was used for preparation. Data from 93 crystals were collected from a single chip, and 14 data sets were rejected in processing. Supplementary Fig. S4 shows the unit cell versus measurement order (order of data collection time), and again there is no evidence of dehydration.

Data collection for hGAC was complicated because of the presence of both orthorhombic and monoclinic crystal forms. The majority of the processable data sets (71 of 137) were of the orthorhombic polymorph, as determined by *XDS*, 33 were monoclinic, and 33 were integrated in *P*1 due to an insufficient number of reflections to determine the crystal system. The unit-cell volume histogram of apo hGAC, shown in Supplementary Fig. S6, shows a bimodal distribution, with the higher population group having double the volume of the lower one. A histogram of the unit-cell volume of the orthorhombic crystals has a narrower unit-cell volume distribution (SD of ∼15%). After filtering with *XSCALE_ISOCLUSTER* 14 data sets were removed, and 57 were used in the final scaling and merging set to give a complete data set with a unit-cell volume SD of 2.7%. Statistics for this data set are given in Supplementary Table S2. The monoclinic and *P*1 data sets were of insufficient quality to scale and merge further. A comparison of this structure with a structure previously determined at 100 K and with drug complexes is forthcoming.

## 5. Discussion and conclusion   

We have demonstrated an integrated system for preparing samples for serial synchrotron crystallography. This system integrates and improves upon previously demonstrated concepts to deliver a versatile, high-performance, low-cost solution. This system has several salient features.

(i) The sample supports are compatible with the existing infrastructure for home-source and mail-in SR crystallo­graphy, including sample-handling tools, storage cassettes/pucks, automounters and goniometer stages. This reduces the overall cost and increases opportunities for data collection.
(ii) A single sample-holder platform accepts diverse sample-support film designs for different size/shape crystals that aid in achieving regular or dispersed crystal positioning.
(iii) Imaging of microcrystals as small as 1–2 µm over the entire active area of the support, not just in wells, is straightforward using both standard visible-light microscopy and laser-scanned nonlinear excitation modalities due to the very thin polymer windows in each well, the thin polymer walls between wells, easy removal of crystal-contrast reducing surrounding liquid, and the weak TPEF and zero SHG signals of polyimide.
(iv) With one or two stages of humidity control provided by the humidity-controlled sample-loading station and the humidified glovebox, dehydration of crystallization drops and crystals, which is a critical issue when loading microcrystals for room-temperature data collection, can be eliminated. As-grown crystal isomorphism is maintained, scaling and merging of diffraction data from many crystals is improved, and the number of crystals required for structure determination is reduced.
(v) Foot-pedal-modulated suction within a humidified environment gives effective control of the solution and crystal flows needed to achieve desired crystal positioning (for example random dispersion or at regularly spaced holes). Positioning over holes can also be achieved by growing crystals *in situ* using vapor diffusion.
(vi) Crystal-position control is achieved using only thin polyimide films with micropatterned features only ∼5–20 µm in height. Wells/walls defined by thick (130–250 µm) substrates (for example silicon and polycarbonate) are not necessary. Background X-ray scatter is low at all positions on the sample support. Diffraction data can be collected from essentially all crystals on the support, not just those located over holes or windows, increasing the efficiency of crystal use. X-rays can be incident from and diffracted through a wide angular range without encountering the support frame. Large sample-support rotations eliminate data-collection issues caused by preferential crystal orientation, and complete data sets can be collected from suitably large individual crystals.
(vii) For room-temperature data collection, ∼4 µm Mylar sealing films provide adequate protection against dehydration for data collection on a timescale of ∼1–2 h. For room-temperature storage and shipping, sealed samples can be stored with reservoir solution in vials, Eppendorf tubes and multiwell plates.
(viii) Crystal cryoprotection is simplified and made more effective. Cryoprotectant-containing solutions (or oils) can be deposited on crystals on the sample support and then withdrawn using suction through the holes in the support; repeating this deposition and removal two or more times can efficiently remove all solvent initially present on the crystal surface, thus eliminating the primary source of ice diffraction in cryocrystallography (Parkhurst *et al.*, 2017 ▸; Moreau *et al.*, 2021 ▸) and minimizing background scatter from excess cryoprotectant.
(ix) For cryogenic temperature data collection, samples can be directly plunged into liquid nitrogen and stored, shipped and handled in the same way and using the same tools as standard loops in cryocrystallography.
(x) The supporting polyimide film is much thinner and has much less thermal mass per unit area than standard ∼25 µm thick cryoEM grids. If ∼1–2 µm crystals are used and excess solution is carefully removed, cooling rates well in excess of 50 000 K ^−1^ (Kriminski *et al.*, 2003 ▸; Warkentin *et al.*, 2006 ▸), which are within a factor of ∼10 of those achieved in typical cryoEM practice (>250 000 K ^−1^), should be achievable using the current state-of-the-art liquid-nitrogen-based cooling methods. Consequently, capture via thermal quenching of room-temperature biomolecular conformations should be nearly as effective using protein microcrystals as when using protein solutions on cryoEM grids (Kaledhonkar *et al.*, 2019 ▸).

How do the present sample supports and protocols for SSX relate to ‘conventional’ crystallography practice using nylon loops and microfabricated loops/mounts? Unlike for loop-mounted crystals, there is no ‘fishing’: multiple crystals are simply deposited (with their mother liquor and any protein or PEG skins) on the support film. Excess liquid is easily removed, background scatter is reduced and cooling rates are increased. Crystals are confined to a plane adjacent to the support film, simplifying imaging and reducing the chance that the X-ray beam illuminates more than one crystal. Cryoprotection is conveniently performed on the support, with little risk of crystal damage or loss. The only disadvantage of the SSX supports is a restricted range of rotation angles due to impingement of the incident beam on the frame of the support. However, the available rotation range is more than adequate for collecting complete data sets from most (suitably sized) single crystals.

For *in situ* crystallization, the present sample supports have an advantage over some goniometer-base-mounted crystallization devices in being fully compatible with beamline automounters and other high-throughput hardware. The present system also allows mother liquor to be suctioned off after crystallization without the risk of crystal damage or dehydration to reduce background scatter and simplify cryocooling.

The integrated sample-handling and mounting system described here provides a comprehensive and highly flexible solution for serial synchrotron crystallography. Moreover, because sample preparation and handling is simplified, sample damage and dehydration are minimized, sample imaging is improved, background scatter is reduced, cryoprotection is simplified and cooling rates are maximized, this system has an excellent potential to replace conventional loops and mounts and associated protocols in a large fraction of biomolecular crystallography applications. Structure determinations, especially at room temperature where crystallography maintains a major advantage relative to cryoEM, typically require not just one crystal but many and that the crystals be highly isomorphous. The present integrated system is optimized for this task and will allow a much more efficient use of crystals and synchrotron beamtime.

## 6. Related literature   

The following references are cited in the supporting information for this article: Darmanin *et al.* (2016 ▸), Grzesiak & Matzger (2008 ▸), Haupert *et al.* (2012 ▸), Haupert & Simpson (2011 ▸), Huang *et al.* (2018 ▸), Newman *et al.* (2016 ▸), Sidorenko *et al.* (2019 ▸) and Wampler *et al.* (2008 ▸).

## Supplementary Material

Supplementary Methods, Supplementary Figures and Supplementary Tables. DOI: 10.1107/S2059798321001868/wa5130sup1.pdf
