# Supplementary material for: Integrated sample-handling and mounting system for fixed-target serial synchrotron crystallography
Source: Acta Crystallogr D Struct Biol. 2021 Apr 19;77(Pt 5):628–44. doi: 10.1107/S2059798321001868 (PMC8098472; doi:10.1107/S2059798321001868)
Supplement: Supplementary file 1 [file d-77-00628-sup1.pdf]

STRUCTURAL
BIOLOGY

Volume 77 (2021)

Supporting information for article:

Integrated sample-handling and mounting system for fixed-target serial synchrotron crystallography

Gabrielle Illava, Richard Jayne, Aaron D. Finke, David Closs, Wenjie Zeng, Shawn K. Milano, Qingqiu Huang, Irina Kriksunov, Pavel Sidorenko, Frank W. Wise, Warren R. Zipfel, Benjamin A. Apker and Robert E. Thorne

Supporting information

S1. Crystallization Methods.

Conditions were identified that yielded roughly uniform size crystals in a low-symmetry space group ($P2_1$), useful for evaluating the effects of oscillation in SSX.(Wierman *et al.*, 2019) Stock solution of FAcD was prepared and purified as described previously.(Wierman *et al.*, 2019) Initial FAcD crystals were obtained using the hanging-drop method using equal volumes of 0.5 m*M* (33 g ml^{-1}) FAcD and a well solution containing 18-22% PEG 3350, 100 m*M* Tris–HCl pH 8.5, and 200 m*M* $CaCl_2$. Large FAcD crystals were used with a Hampton Research seed bead kit (HR2-320) to prepare seed stock. FAcD crystals with approximate dimensions of 10 × 10 × 40 μm^3 to 20 × 20 × 60 μm^3, suitable for SX, were obtained via hanging drop vapor diffusion with drops comprised of a 2:1:3 ratio of 18-22% PEG 3350, 100 m*M* Tris–HCl pH 8.5, and 200 m*M* $CaCl_2$ well solution (1 µL), seed stock (0.5 µL) to 18 mg ml^{-1} protein solution (1.5 µL). The rod shape of these crystals was useful in evaluating preferential orientation effects on different SSX support designs.

Two different crystal forms of HEWL – tetragonal and orthorhombic – were prepared. For the tetragonal form, HEWL powder (VWR, 18J2556261 CAS 12650-88-3) was dissolved in 0.5 M acetic acid pH 4 to obtain a 100 mg mL^{-1} solution. Large crystals were obtained via batch growth in a 5 mL Eppendorf tube by mixing 1 mL of protein solution and 3 mL of precipitant solution [20% (*w*/*v*) NaCl, 6% PEG 6 K, 500 m*M* NaOAc pH 4.0]. Crystals of typical size 10 × 10 × 10 μm^3 were obtained after 24 hours at 4° C. The solution in the tube was then slowly replaced by a storage solution [8% (*w*/*v*) NaCl, 100 m*M* NaOAc pH 4.0] over the course of three days. The crystals grew to a final size of 40 × 40 × 40 μm^3 at 4°C.

Conditions to produce tetragonal HEWL "microcrystals" (crystals with volumes of less than 5 × 5 × 5 μm^3) were adapted from Darmanin *et al.*(Darmanin *et al.*, 2016). 40 µL of 160 mg mL^{-1} HEWL in 500 mM acetic acid pH 4.0 was added to 170 µL precipitant [18% (*w*/*v*) NaCl, 6% PEG 6 K] in an Eppendorf tube and vortexed. Crystals formed immediately. Crystal size was reproducibly controlled by adjusting the amount of protein solution added to the 170 µL of precipitant solution: 40 µL of protein solution gave ~ 20 × 20 × 20 μm^3 crystals, 20 µL of protein solution gave ~ 10 × 10 × 10 μm^3 crystals, and 5 µL gave ~2 × 2 × 2 μm^3 crystals, respectively.

Orthorhombic HEWL crystals of size ~ 50 × 50 × 50 µm³ were obtained via batch growth in Eppendorf tubes using a solution containing 100 mg ml^{-1} lysozyme, 50 m*M* NaOAc pH 4.5, and 25% (*w*/*v*) NaCl. The orthorhombic lattice was confirmed both by the detection of a second harmonic generation (SHG) signal in laser scanned imaging (absent for the tetragonal crystal form) and by x-ray diffraction (Grzesiak & Matzger, 2008; Wampler *et al.*, 2008; Haupert *et al.*, 2012).

Human Glutaminase C is a key enzyme in cancer cell glutamine metabolism and a potential therapeutic target (Huang *et al.*, 2018). Solutions of 20 mg/mL hGaC (in 50 mM NaCl and 5 mM Tris-HCl, pH 7.5) and 30 mM inhibitor (in DMSO) were prepared. The protein– inhibitor complexes were formed by mixing 95 µL of the GaC solution and 5 µL of the inhibitor solution, yielding a mole ratio of 1:4, and then incubating the on ice for 1 h. Crystals were grown at 20 °C by the hanging drop vapor diffusion method in crystallization trays. Typically, 1.2 µl of the complex solution was mixed with 1.2 µl of a reservoir solution consisting of 10% PEG6000 (w/v), 1.0 M LiCl, and 0.1 M Tris-HCl buffer (pH 8.5). Crystals were observed within 24 h, reaching an average size of 100 µ × 100 × 200 µm^3 after 7 days. GaC-I crystals were also grown in sealed petri dishes in both hanging and sitting drop configurations by loading the complex + reservoir solution directly onto the sample support film and suspending the sample support above reservoir solution.

FAcD crystals were also prepared cryogenic data collection. Crystals grown in 2.5 µL drops on coverslips were resuspended using 10 µL of well solution mixed with 10 µL of a 40% glycerol solution. 15 µL of this crystal solution was then loaded onto the sample support and excess liquid removed by vacuum and/or blotting, all within the humidified sample loading station / humidified enclosure as for room temperature. The samples were then transferred using a custom wand to the sample plunge stage of a Nanuq™ automated hyperquenching cryocooler. The Nanuq™ and adjacent work area were enclosed in a tent with air humidified to 80% r.h. to reduce dehydration. Samples were plunge cooled and automatically stored in UniPucks, and the pucks were then transferred to storage dewars.

S2. Beamline setup

Data from FAcD crystals were first collected at T=100 K at the XF17ID2 (FMX) beamline at NSLS-II, and then at room temperature at the ID7B2 (FlexX) beamline at CHESS. Data from lysozyme and hGAC crystals were collected at room temperature at ID7B2.

At FMX, an incident X-ray flux 1.1 × 10^{12} ph/s at wavelength 0.979 Å (12.66 keV) was delivered in a 10 µm × 10 µm (v × h FWHM) beam, and diffraction recorded on an EIGER 16M detector.

At ID7B2, incident X-rays at wavelength 1.127 Å (11 keV) was delivered in a 12 µm × 9 µm (v × h FWHM) beam, and diffraction recorded on an EIGER 1M detector. In Fall 2019 and early 2020 experiments on FAcD and lysozyme crystals at room temperature, the incident X-ray flux was 1.6 × 10^{11} ph/s and 2.0 × 10^{11} ph/s, respectively. In June 2020 experiments on hGaC, the incident flux increased to 9.8 × 10^{11} ph/s.

S3. Crystal imaging using laser scanning microscopy

For imaging crystals embedded in precipitate, protein or PEG skins, LCP, and other strongly-scattering and/or high refractive index media, laser-scanning nonlinear optical imaging modalities (two-photon excitation fluorescence (TPEF) and second harmonic generation SHG) have significant advantages (Kissick *et al.*, 2011; Haupert & Simpson, 2011; Newman *et al.*, 2016; Padayatti *et al.*, 2012). Crystals on sample supports were imaged at Cornell University using a laser scanning microscope incorporating a new femtosecond pulse laser source (Sidorenko *et al.*, 2019). As shown in Fig. S2, for crystals on sealed sample supports that were kept “wet”, reducing ordinary optical image contrast, the nonlinear methods give superior imaging. Weak TPEF generated by the polyimide support is strongest at the thicker walls and posts, which can then be used as fiducials in locating crystals.

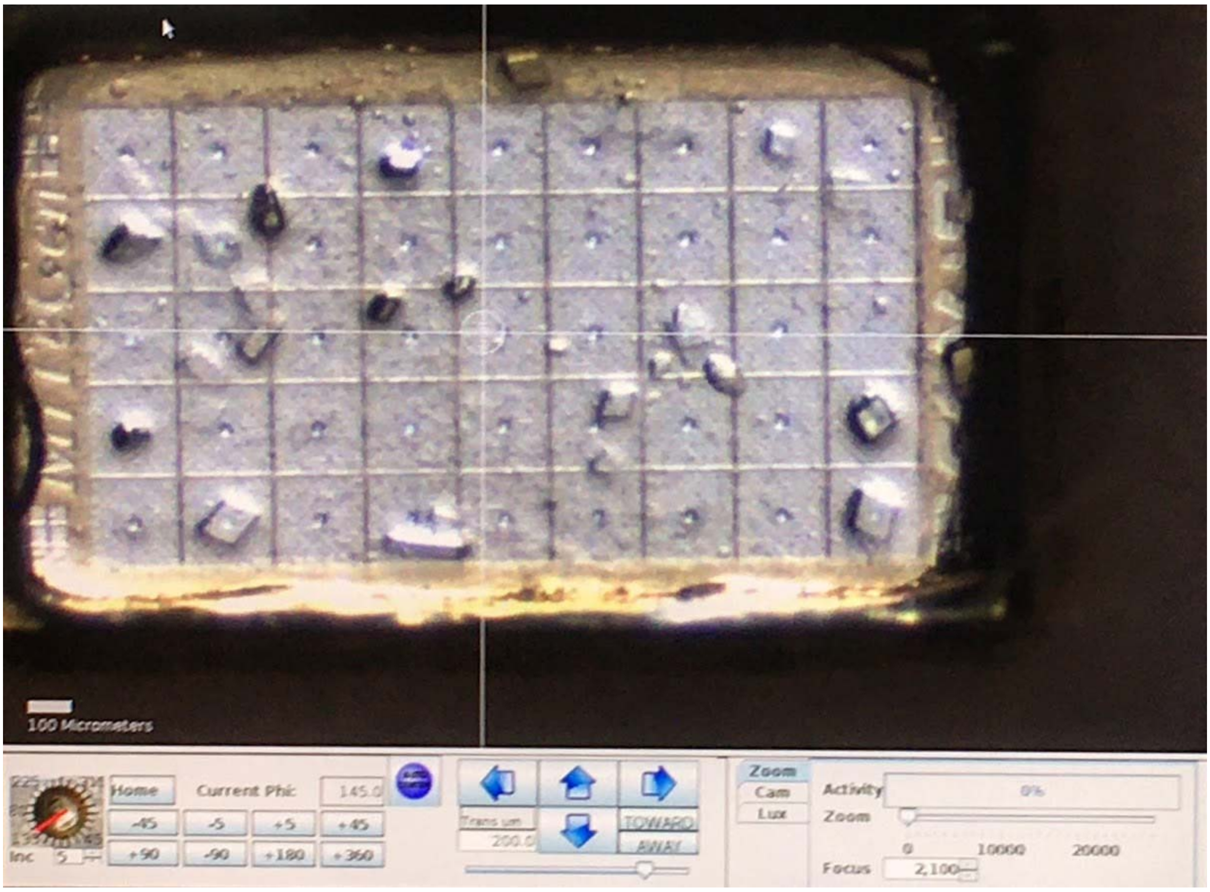

Figure S1. Image recorded at CHESS beamline ID7B2 of GaC-I crystals on a sample support at room temperature, showing excellent contrast achieved when excess liquid is removed.

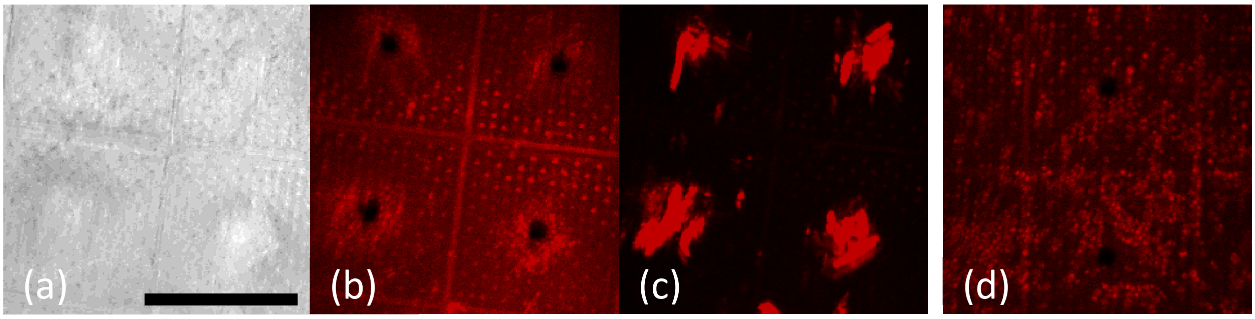

Figure S2 (a) Laser transmission, (b) TPEF, and (c) SHG images of FAcD crystals on an SSX sample support, each cell of which has a central hole and an array of posts (Fig. 2(i)). The smallest crystal dimension visible is ~5 µm. (d) TPEF image of ~2-3 µm tetragonal lysozyme crystals, which generate extremely weak second harmonic signal. Weak polyimide fluorescence allows visualization of thicker portions of the film that provide coordinate references for locating crystals. The frame size in each image is 400 µm and the scale bar is 200 µm.

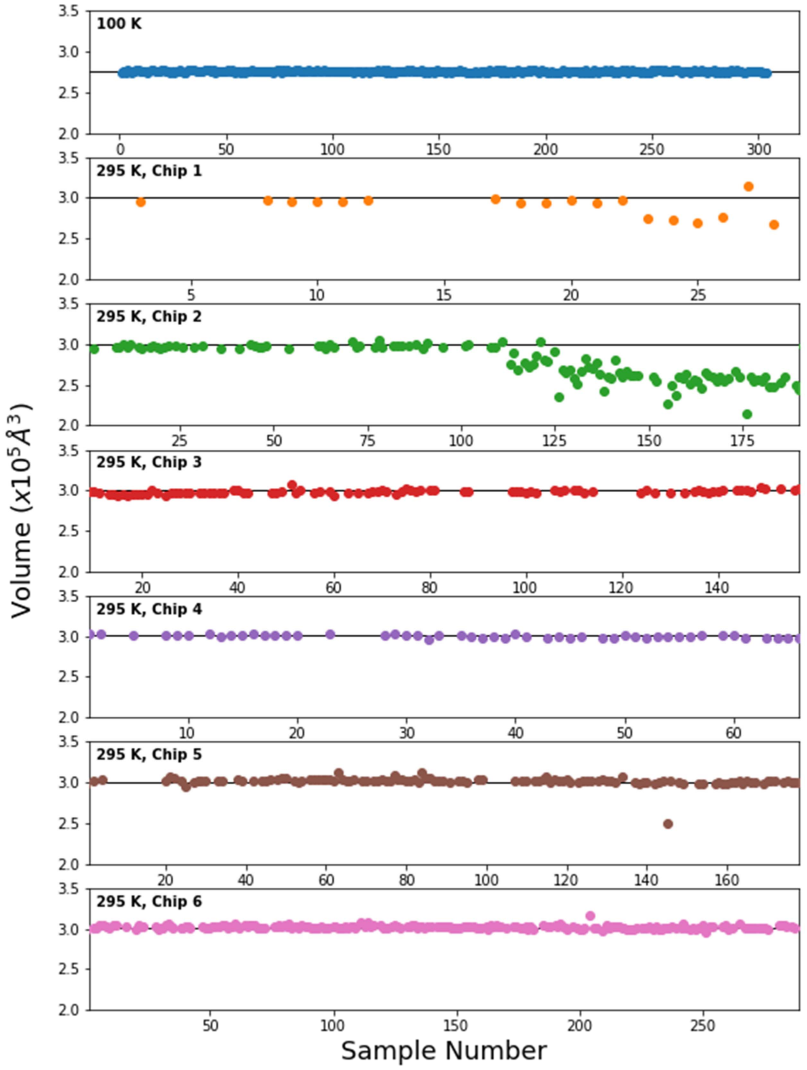

Figure S3 Unit cell volume measured for FAcD crystals on a single support, in order of measurement (and thus in order of data collection time) at *T*=100 K and *T*=295 K. Horizontal lines indicate the mean unit cell volume for each temperature. At 295 K, twinned crystals that displayed pseudoorthorhombic symmetry were automatically removed. Chip 1 at *T*=295 K was loaded at 70% humidity. Chip 2 at *T*=295 K was unsealed during data collection, leading to sample dehydration which can be clearly seen in the decreasing unit cell volume.

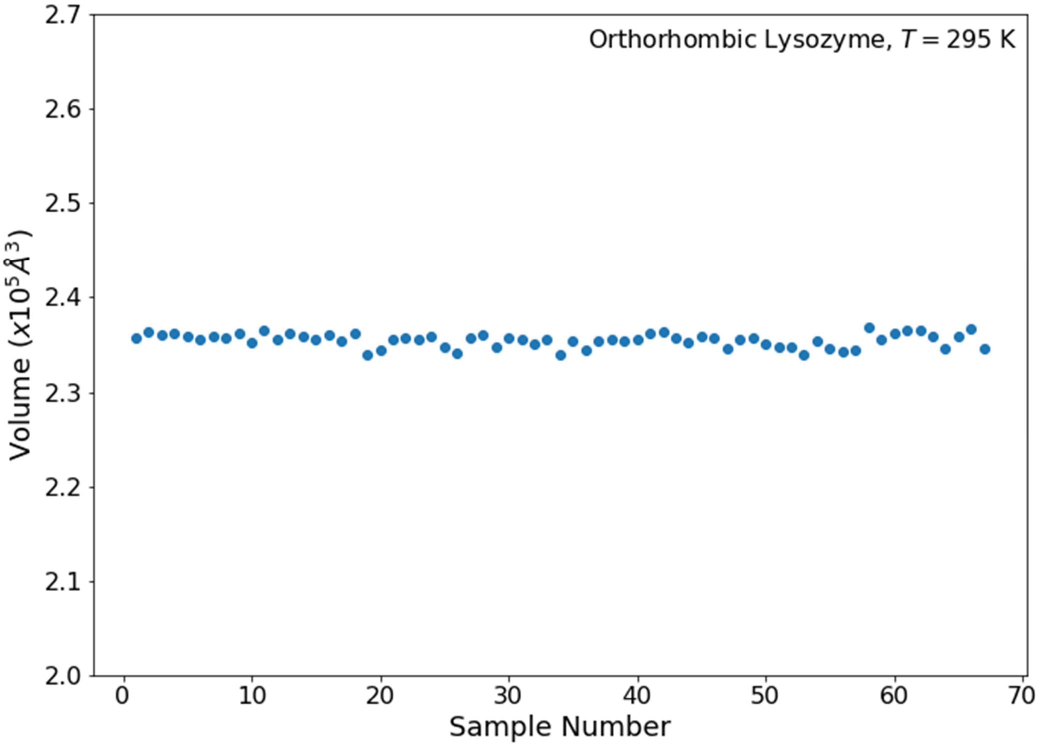

Figure S4 Unit cell volume measured for orthorhombic lysozyme crystals on a single support, in order of measurement (and thus in order of data collection time) at T=295 K. The unit cell remained nearly constant, indicating no appreciable dehydration occurred during data collection.

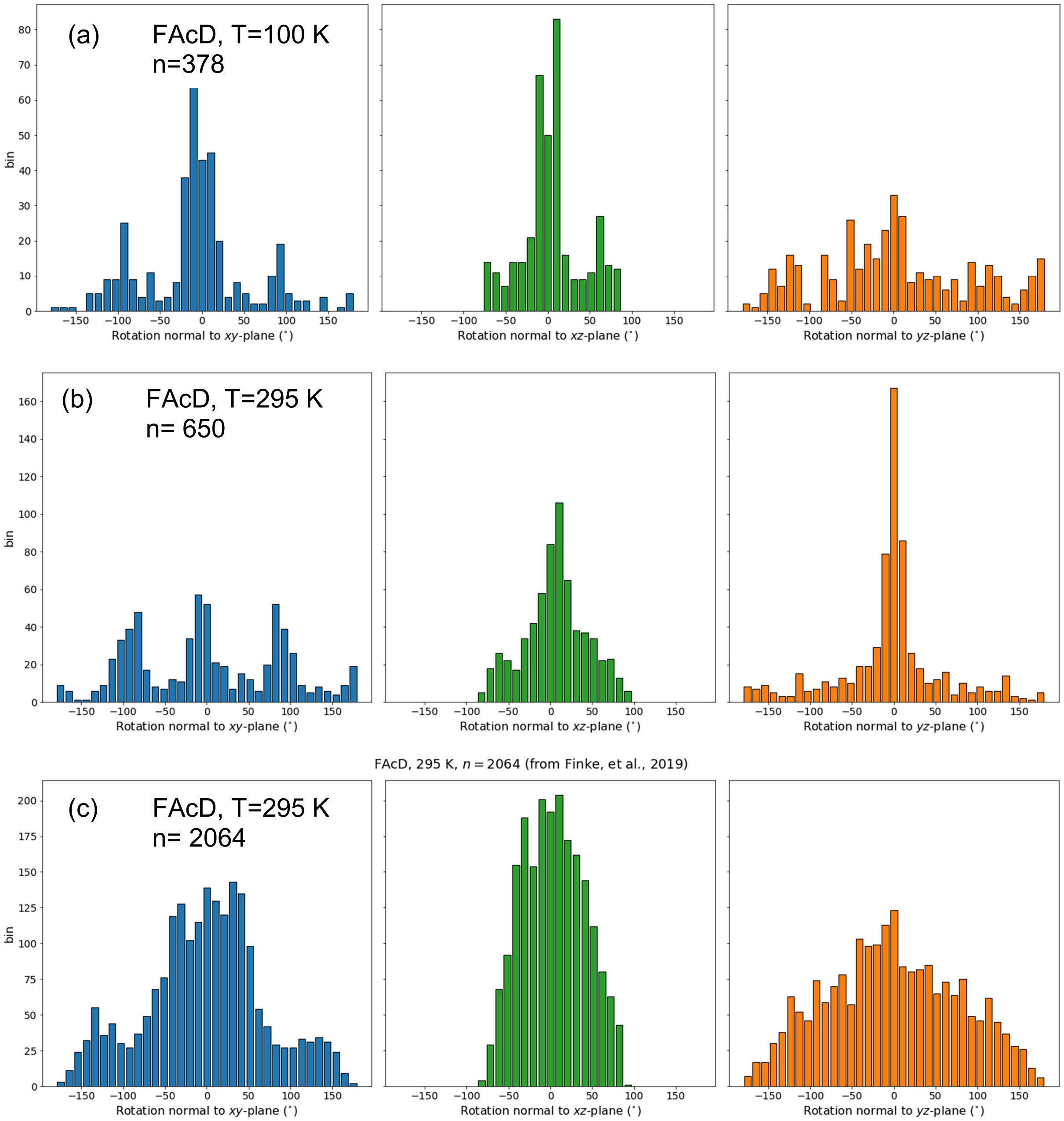

Figure S5 Distribution of FAcD crystal orientations on sample supports as in Fig. 5(a) as determined by XDS at (a) T=100 K and (b) T=295 K. (c) Corresponding distribution of FAcD crystal orientations on fixed target silicon well chips in Wierman et al. (Wierman *et al.*, 2019).

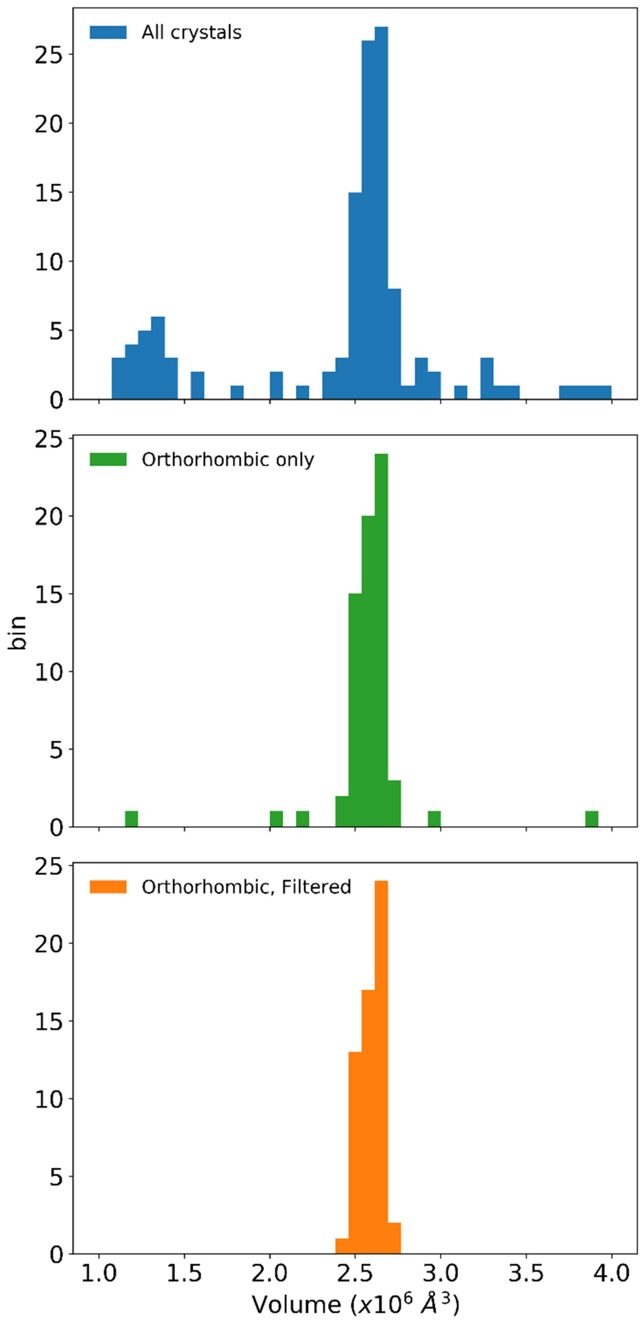

Figure S6 Distribution of unit cell volumes for apo-hGaC crystals at room temperature determined using XDS. (a) Cell volume distribution for all 137 processable crystals. (b) Cell volume distribution for 71 crystals with orthorhombic unit cells. (c) Cell volume distribution after filtering with XSCALE_ISOCLUSTER, for 57 crystals with orthorhombic cells.

Table S1 Dimensions of the SSX sample support.

<table>
<tr><th></th><th>Width</th><th>Length</th><th>Thickness</th></tr>
<tr><td>Frame</td><td>2.5 mm</td><td>17.8 mm</td><td>0.5 mm</td></tr>
<tr><td>Frame aperture</td><td>1.5 mm</td><td>5.25 mm</td><td>-</td></tr>
<tr><td>Sample support film</td><td>2.5 mm</td><td>3.5 or 6.5 mm</td><td>10 or 20 µm</td></tr>
<tr><td>Sealing film</td><td>2.5 mm</td><td>9.0</td><td>3.2 µm</td></tr>
<tr><td colspan="2">Distance from flat bottom of base to end of frame</td><td colspan="2">18.7 mm</td></tr>
<tr><td colspan="2">Distance from flat bottom of base to center of frame aperture</td><td colspan="2">15.6 mm</td></tr>
</table>

Table S2

	FAcD-100 K	FAcD-room temperature	Orthorhombic Lysozyme	Apo-hGAC
Beamline	17-ID-2 (NSLS-II)	ID7B2 (CHESS)	ID7B2 (CHESS)	ID7B2 (CHESS)
Number of crystals	258	166	79	57
Temperature (K)	100	295	295	295
Wavelength (Å)	0.9794	1.127	1.127	0.9686, 1.127
Resolution range (Å)	40.53 - 1.39 (1.44 - 1.39)	19.43 - 2.07 (2.144 - 2.07)	34.06 - 2.0 (2.072 - 2.0)	48.27 - 3.003 (3.11 - 3.003)
Space group	P 1 21 1	P 1 21 1	P 21 21 21	P 21 21 21
Unit cell	41.6 79.1 83.8 90 103 90	43.16 82.28 88.04 90 103.015 90	37.79 78.59 78.62 90 90 90	103.16 138.62 177.39 90 90 90
Total reflections	2446524 (181109)	535426 (48389)	216265 (20339)	512646 (46953)
Unique reflections	105667 (10539)	36376 (3453)	16400 (1606)	50938 (3639)
Multiplicity	23.2 (17.2)	14.7 (13.4)	13.2 (12.7)	10.1 (9.3)
Completeness (%)	99.55 (99.41)	99.38 (95.20)	99.59 (99.19)	95.36 (71.36)
Mean I/sigma(I)	13.72 (1.45)	5.00 (0.37)	13.73 (3.24)	2.76 (0.16)
Wilson B-factor	14.44	35.85	33.94	87.51
R-merge	0.2228 (4.68)	0.4501 (19.69)	0185 (2.76)	0.7607 (-18.01)
R-meas	0.2283 (4.84)	0.4651 (20.48)	01929 (2.88)	0.7994 (-19.01)
R-pim	0.04453 (1.114)	0.11 (5.274)	0.05234 (0.7926)	0.2319 (-5.776)
CC1/2	0.997 (0.267)	0.979 (0.105)	0.986 (0.618)	0.97 (0.109)
CC*	0.999 (0.649)	0.995 (0.436)	0.997 (0.874)	0.992 (0.443)
Reflections used in refinement	105661 (10535)	36409 (3453)	16372 (1600)	49403 (3631)
Reflections used for R-free	5275 (516)	1821 (172)	818 (81)	2458 (176)
R-work	0.1803 (0.2901)	0.2422 (0.3858)	0.1699 (0.2888)	0.2552 (0.5340)
R-free	0.1908 (0.3068)	0.3111 (0.4045)	0.2195 (0.3768)	0.3272 (0.5141)
CC(work)	0.968 (0.670)	0.930 (0.233)	0.960 (0.763)	0.939 (0.316)
CC(free)	0.973 (0.707)	0.890 (0.156)	0.942 (0.672)	0.827 (0.234)
Number of non-hydrogen atoms	5162	4836	2135	12600
macromolecules	4777	4696	2026	12600
ligands	8	1	4	n/a
solvent	377	139	105	n/a
Protein residues	594	594	262	1616
RMS(bonds)	0.006	0.009	0.006	0.010
RMS(angles)	0.92	1.14	0.91	1.42
Ramachandran favored (%)	96.95	93.22	97.22	86.06
Ramachandran allowed (%)	2.88	6.27	2.78	12.38
Ramachandran outliers (%)	0.17	0.51	0.00	1.56
Rotamer outliers (%)	0.62	0.00	0.00	0.07
Clashscore	5.63	8.33	4.00	14.47
Average B-factor	17.90	37.90	35.05	89.25
macromolecules	17.43	37.87	34.78	89.25
ligands	26.61	37.77	37.10	n/a
solvent	23.58	38.70	40/15	n/a
